# Insulator-based loops mediate the spreading of H3K27me3 over distant micro-domains repressing euchromatin genes

**DOI:** 10.1186/s13059-020-02106-z

**Published:** 2020-08-03

**Authors:** Alexandre Heurteau, Charlène Perrois, David Depierre, Olivier Fosseprez, Jonathan Humbert, Stéphane Schaak, Olivier Cuvier

**Affiliations:** 1 Chromatin Dynamics and Cell Proliferation, Center of Integrative Biology (CBI), Laboratoire de Biologie Moléculaire Eucaryote (LBME), CNRS, Université Fédérale Paul Sabatier de Toulouse (UPS), F-31000 Toulouse, France; 2 St. Patrick Research Group in Basic Oncology, Laval University Cancer Research Center, Centre Hospitalier Universitaire de Québec City, Quebec, QC G1R 3S3 Canada

**Keywords:** Insulators, Higher-order chromatin folding, Topologically associating domains, Chromosome compartmentalization into euchromatin and heterochromatin domains

## Abstract

**Background:**

Chromosomes are subdivided spatially to delimit long-range interactions into topologically associating domains (TADs). TADs are often flanked by chromatin insulators and transcription units that may participate in such demarcation. Remarkably, single-cell Drosophila TAD units correspond to dynamic heterochromatin nano-compartments that can self-assemble. The influence of insulators on such dynamic compartmentalization remains unclear. Moreover, to what extent heterochromatin domains are fully compartmentalized away from active genes remains unclear from Drosophila to human.

**Results:**

Here, we identify H3K27me3 micro-domains genome-wide in Drosophila, which are attributed to the three-dimensional spreading of heterochromatin marks into euchromatin. Whereas depletion of insulator proteins increases H3K27me3 spreading locally, across heterochromatin borders, it concomitantly decreases H3K27me3 levels at distant micro-domains discrete sites. Quantifying long-range interactions suggests that random interactions between heterochromatin TADs and neighbor euchromatin cannot predict the presence of micro-domains, arguing against the hypothesis that they reflect defects in self-folding or in insulating repressive TADs. Rather, micro-domains are predicted by specific long-range interactions with the TAD borders bound by insulator proteins and co-factors required for looping. Accordingly, H3K27me3 spreading to distant sites is impaired by insulator mutants that compromise recruitment of looping co-factors. Both depletions and insulator mutants significantly reduce H3K27me3 micro-domains, deregulating the flanking genes.

**Conclusions:**

Our data highlight a new regulatory mode of H3K27me3 by insulator-based long-range interactions controlling distant euchromatic genes.

## Introduction

Chromatin folding in 3D has been revealed through microscopy [1, 2] and genome-wide chromosome conformation capture methodologies (3C/Hi-C [3];) [4], which eventually highlighted how chromosomes fold into topologically associating domains (TADs) and sub-TADs [5–12] [13–15]. TADs notably promote specific long-range contacts between distant sites and regulatory elements that localize within the same topological unit. At higher resolution, smaller TADs may further delimit cell-type-specific long-range contacts, thus contributing to cell-type-specific gene expression programs [6, 9, 16–22].

Hi-C contact matrices show that frequencies of long-range interactions (LRIs) largely depend on distances, as explained by polymer physics models [14, 23]. LRIs highlight how TADs are insulated from neighboring domains due to either self-assembly properties of TADs or to their delimitation by “insulators/boundaries” [12, 20, 24–27]. TAD insulators may restrict LRIs with sites localized in adjacent TADs. Accordingly, removal of a TAD border results in gene deregulation. This may involve long-range contacts between a gene and the regulatory sequences localized in the adjacent TAD [9, 28, 29]. The existence of TADs and domains may however not solely rely on borders but also on intrinsic self-assembling/propagation properties, e.g., shown for polycomb repressive complexes (PRC1 or 2) in inactive TADs [1, 15, 18, 30–33], or of transcription factors in context of active TADs [34] along with epigenetic mechanisms involving non-coding RNAs, DNA methylation, or post-translational modifications (PTMs) of histones [7, 14, 35–38]. 3D clustering of factors may then lead to “phase separation” involving multimerized DNA-protein complexes or liquid droplet [31, 34, 39], possibly accounting for the maintenance of gene expression programs [30, 40]. High-resolution mapping of TADs in Drosophila highlights their good correspondence with repressed domains including at levels of highly resolved loop-based (sub-)TADs [16, 20, 21]. Strikingly, repressive TADs are dynamic structures defining nano-compartments visible in single cells [1, 15]. How much epigenetically marked repressed TADs maintain their identity depending on self-maintenance or on TAD borders remains unknown.

Of interest, TAD borders fall into sites recognized by a family of factors called insulator proteins that notably include CCCTC-binding factor (CTCF) [41, 42]. Additional insulator proteins are being identified defining a growing family of factors from Drosophila to human [43]. Major factors include CTCF, GAGA-binding factor (GAF) [44], M1BP [20, 45], and Boundary Element-Associated Factor of 32 KDa (Beaf32) [46]. Insulator proteins “insulate” a (*trans*-)gene from its environment [47] and from activation by promiscuous enhancers [48]. Insulating activity relies on interactions with co-factors including cohesin or CP190 to stabilize loops through evolutionary conserved principles [19, 22, 25, 26, 49–51]. Remarkably, inversion of CTCF sites impairs genome topology and enhancer-promoter long-range contacts [28, 52]. Insulator-based regulation of long-range contacts may contribute to link transcriptional programs to 3D folding, as shown upon stem cell differentiation [6, 9, 19, 24, 37].

Insulators further act as chromatin barrier insulators (CBI) [53] that participate in Hox-based para-segment identity in flies [54, 55]. CTCF (and other insulator protein) sites are specifically enriched at heterochromatin domain borders [42, 46, 56–58]. In Drosophila, borders lacking dCTCF harbor other types of insulator proteins whose binding is required to block the spreading of repressive histone marks, including histone H3 trimethylated on -lysine 9 (H3K9me3) [46] and on -lysine 27 (H3K27me3) [59, 60]. Removal of insulators may not systematically lead to spreading defects for all borders. Moreover, the role of insulators is unclear with respect to the highly dynamic nature of TAD compartments in single cells [1, 15], suggesting possible interactions between H3K27me3 domains and the flanking euchromatin.

Here, we analyzed the spreading of heterochromatin H3K27me3 marks depending on insulator proteins and long-range interactions (LRIs) by analyzing Hi-C data [16, 20, 21] aggregated onto TAD borders. Removal of insulator proteins Beaf32 leads to H3K27me3 spreading locally, across borders. In addition, Beaf32 promotes spreading onto distant euchromatin sites named “micro-domains.” Systematic measurements of LRIs suggest that H3K27me3 micro-domains do not form due to the weakness of TAD borders. Rather, micro-domains were visible at sites showing high levels of LRIs, including distant dCTCF and GAF insulator sites bound by the looping co-factor CP190. Also, micro-domain formation appears to depend on such specific insulator-mediated LRIs utilized to spread H3K27me3 to distant sites through looping. Supporting these results, specific synthetic mutants that impair LRIs compromise distant spreading over micro-domains. Distant spreading at micro-domains is further associated with insulator-based control of genes and it influences H3K27me3 throughout developmental stages of Drosophila. Our data highlight how specific LRIs encoded by insulator-mediated loops contribute to the regulation of H3K27me3 spreading over the distance. We propose that micro-domains reflect how insulators participate to chromatin folding dynamics in 3D, aside additional factors required to separate heterochromatin nano-compartments from nearby euchromatin domains.

## Results

### H3K27me3 micro-domains are associated with dCTCF and GAF insulator binding sites

**Fig. 1:**
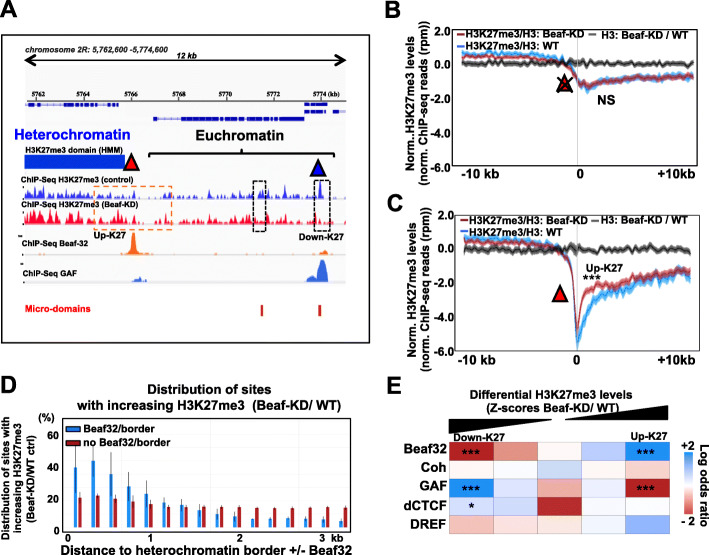
The chromatin insulator protein Beaf32 protects genes from H3K27me3 spreading at borders. **a** Genomic view of our ChIP-seq data for H3K27me3 reads (*y*-axis) in S2 cells depleted for the insulator protein Beaf32 (“Beaf-KD”) or in control, siRNA-treated wild-type cells (“WT” control). The orange and blue triangles represent Beaf32 and GAF binding sites, respectively, as detected by ChIP-seq (see “Methods”). The blue bar represents a large H3K27me3 heterochromatin domain as detected by hidden Markov model (HMM; see “Methods”) often bordered by binding sites of insulator proteins such as Beaf32. Note that Beaf32 binding sites are enriched at the borders of H3K27me3 domains (930 sites; Fisher exact test, *p* value < 1e−151) (see panel **c**). The red bars represent “micro-domains” of H3K27me3 (see text for details). The dashed rectangles in black highlight the corresponding regions where such H3K27me3 levels may decrease in Beaf-KD compared to WT control, contrasting with the apparent increase in H3K27me3 levels near borders flanked by a Beaf32 site (see dashed rectangle in orange; see also panels **b** and **c**). **b**, **c** Averaged H3K27me3 levels centered surrounding the H3K27me3 domain borders (see “Methods” for details) in absence or in presence of a Beaf32 site (panels **b** and **c**, respectively) or a Beaf32 site flanking heterochromatin domains. The asterisks indicate a significant *p* value (Wilcoxon pairwise test, *p* value < 1.e−4) for the statistical difference in H3K27me3 levels (in the 0–4 kb euchromatin segments next to heterochromatin), between Beaf-KD compared to WT control cells (**c**) compared to borders without Beaf32 sites (“NS”; not significant). **d** Distribution of sites (bins) with increasing H3K27me3 levels relative to heterochromatin borders (*x*-axis, position 0) with Beaf32 (blue bars) or without (red bars). Note that Beaf32 specifically deregulates H3K27me3 at sites flanking repressive H3K27me3 heterochromatin flanked by a Beaf32 binding site. The error bars represent the variability of the signal from independent replicates with all bins in the indicated intervals (see “Methods”). **e** Genome-wide analysis representing the relative enrichments of genes associated with H3K27me3 variations scored in their TSSs (± 1 kb), depending on binding of insulator proteins (Beaf32, Cohesin, GAF, dCTCF, DREF) in the same windows (TSSs ± 1 kb). All TSSs were systematically ranked according to variations between Beaf-KD and control cells of H3K27me3 levels (TSSs ± 1 kb) (see “Methods”). Log odds ratios were then calculated for all genes for ranking them in quintiles. Enrichment tests were performed by intersecting such quintiles (groups of genes ranked depending on H3K27me3 variations ± 1 kb surrounding TSS) with TSSs with or without insulator protein sites in the same interval (± 1 kb TSS) (see also Additional file 1: Fig. S1F). The indicated *p* values (asterisks) were calculated using Fisher’s exact test in presence as compared to absence of sites

Insulator proteins often bind to sites flanking heterochromatin domains from Drosophila to human [46, 56], as illustrated for Beaf32 (Fig. 1a). In such contexts, removal of Beaf32 was accompanied with the downregulation of adjacent genes due to heterochromatin spreading [46, 59]. Increasing levels of H3K27me3 levels could be detected near Beaf32 sites flanking heterochromatin (Fig. 1a–c). Systematic measurements showed that Beaf32 depletion (“Beaf-KD”) led to a relatively modest yet significant increase in H3K27me3 levels as compared to siRNA-treated control cells (Fig. 1b, c; *p* value of 1e−4) (Additional file 1: Fig. S1A). Such increase was specific of heterochromatin domains with a Beaf32 site as compared to control domains without a Beaf32 site. Of note, the increase was not detected for total histone H3 reads (Fig. 1b, c), indicating that it is specific of the H3K27me3 mark. Furthermore, such increase in H3K27me3 levels was preferentially associated with genes being downregulated upon Beaf-KD, unlike control or upregulated genes (Additional file 1: Fig. S1B) [59]. Furthermore, genes encoding the subunits of Polycomb repressor complex showed no variation in expression upon such depletion of insulator proteins (Additional file 1: Fig. S1E), arguing against indirect defects in regulating H3K27me3 at least due to PRC2 deregulation. Actually, the distribution of H3K27me3 spreading upon Beaf32 depletion was detected specifically for heterochromatin borders flanked by Beaf32 sites as shown (Fig. 1d), thus confirming a specific defect in spreading.

Our results showed that the influence of Beaf32 was not drastic, raising the possibility that additional factors may be required to block heterochromatin. Since two distant insulators can interact to form a loop, we sought to test if two insulators could better block H3K27me3 spreading. However no difference in spreading was detected depending on the presence of one or two insulators bracketing the domain (Additional file 1: Fig. S1D; see below). Alternatively, the moderate spreading of H3K27me3 could suggest a requirement for additional factors that participate in blocking heterochromatin. Since > 91% of Beaf32 sites co-localize (± 1 kb) with TSSs, we sought to better evaluate the influence of Beaf32 or of other factors by taking assessing H3K27me3 in an otherwise similar genomic context, ± 1 kb of TSSs (see “Methods”). A systematic scoring of H3K27me3 variations between Beaf32-depleted cells compared to wild-type control confirmed that in this context, Beaf32 sites was the insulator proteins that was specifically associated with increasing levels of H3K27me3 (Fig. 1e). Of interest, the opposite effect—i.e., the decrease in H3K27me3 levels upon depletion of Beaf32 compared to control cells—was detected at certain insulator factor sites including GAF and to a lesser extent dCTCF sites (Fig. 1a, e). These results were also confirmed when scoring variations in H3K27me3 surrounding all insulator sites, independently of TSSs (Additional file 1: Fig. S1F).

**Fig. 2:**
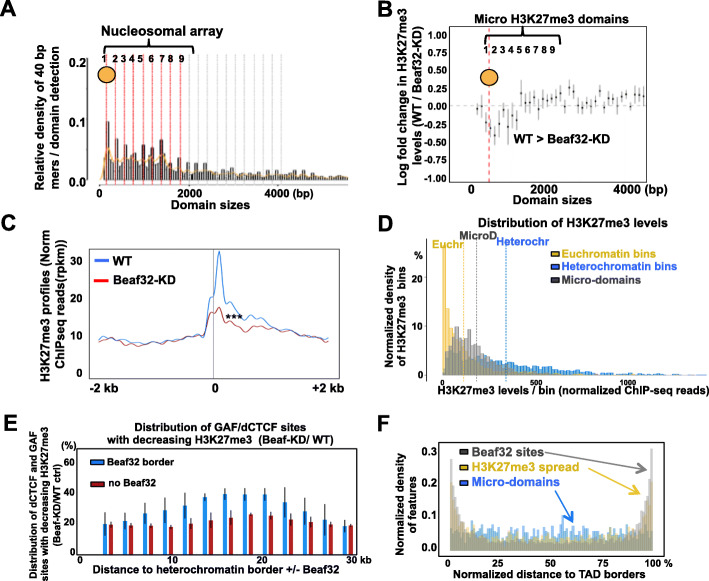
Micro-H3K27me3 domains are altered upon insulator protein depletion. **a** Distribution plot showing the density of micro-H3K27me3 domains (*y*-axis) depending on the sizes of the domains (*x*-axis), as detected by scoring H3K27me3 levels by scanning the genome with a resolution of 40 bp bin size using NormR (see “Methods” [61];). Micro-domains’ sizes distribute into mers of nucleosome arrays (see dotted lines every 200 bp) thus reinforcing the relevance of the detected signal unlike for swap controls (see “Methods”). The red dotted lines highlight the interval of micro-domain sizes. **b** Plot showing the averaged log ratio of H3K27me3 levels of micro-domains in Beaf-KD as compared to WT control cells, depending on domain sizes (*x*-axis). **c** Average H3K27me3 levels in micro-domains in Beaf-KD as compared to WT control cells. Note that Beaf-KD leads to a significant reduction in H3K27me3 levels as compared to WT cells (***; Wilcoxon pairwise test, *p* value <1e−4; see Additional file 2: Fig. S2D). **d** Distribution plot quantifying the normalized densities of H3K27me3 levels from normalized ChIP-seq reads in bins corresponding to micro-domains compared to randomized control bins of the same distribution sizes selected out of euchromatin or heterochromatin domains (see “Methods”). The dashed lines highlight the mean values of H3K27me3 densities for micro-domains controls as indicated. **e** Distribution of insulator sites (bins) bound by GAF/dCTCF that harbor decreasing H3K27me3 levels. Sites were plotted relative to heterochromatin borders (*x*-axis) with a Beaf32 site (blue bars) or not (brown bars). Note that the influence of Beaf32 borders is detected even for GAF/dCTCF sites localizing over long distances (> 10 kb) from such borders. The error bars represent the variability of signal from independent replicates with all bins in the indicated intervals (see “Methods” for details). **f** Bar plot showing the relative distribution of Beaf32 sites (black), H3K27me3 micro-domains (blue) and sites with increasing H3K27me3 spreading (yellow) within euchromatin domains. All sites were distributed as a function of the positioning with respect to borders (0 and 100 are the two sides of euchromatin domains, and 50% the middle of such domains)

Our above results prompted us to systematically detect regions where decreasing H3K27me3 levels might be detected upon Beaf32 depletion, genome-wide and without a priori. We thus re-analyzed our chromatin immunoprecipitation experiments (ChIP-seq) for H3K27me3 in control or Beaf32-depleted cells and scanned the genome with NormR [61]. Briefly, we scored normalized reads in sliding windows (bins) of 40 bp compared to input and then compared to depleted conditions (see “Methods”). As a result, novel “micro-domains” of H3K27me3 were identified (Fig. 2a). Of note, micro-domains could not be previously detected by classic, e.g., hidden Markov model (HMM) methods, in part because of their relatively small sizes and low H3K27me3 levels (see below). Plotting the density of 40 bp-bins showed a non-random distribution of their lengths, corresponding to nucleosome mers (Fig. 2a, b). Micro-domains corresponded to 2–8 nucleosomes with more than 65% of them of length < 2 kb. Most micro-domains further showed a significant reduction in the log ratio of H3K27me3 levels upon Beaf32 depletion compared to control cells (Fig. 2b). Such a decrease was more significant for micro-domains harboring 2 up to 4 nucleosome mers, as also confirmed by inspecting averaged profiles markedly impaired by the depletion compared to control cells (Fig. 2c; Additional file 1: Fig. S2A; *p* value of 1e−6). The decrease was confirmed by re-measuring H3K27me3 in micro-domains by qPCR (Additional file 1: Fig. S2B-C). The reduction was most significant for 2–4 nucleosome mers with no difference in spreading over 2 kb distances (Additional file 1: Fig. S2D-E). From these results, we defined a list of 1311 H3K27me3 micro-domains of sizes < 2 kb (Additional file 2: Table S1) for all subsequent genomic analyses, of which 722 flanked (< 1 kb) from a TSS (Additional file 3: Table S2). Micro-domains are distinct from known conventional heterochromatin domains as evident by differences in their sizes and intensities, as shown by genome-wide analyses of H3K27me3 levels in micro-domains, heterochromatin or euchromatin for bins of identical sizes (Fig. 2d). This illustrates how euchromatic micro-domains (730 bp average size), i.e., the equivalent of 3–4 nucleosomes, may be distinct from larger/denser and epigenetically stable heterochromatin domains.

Micro-domains could reflect the observed decrease upon Beaf32 depletion of H3K27me3 levels at GAF/dCTCF sites (Fig. 1e). Analyzing the distribution of such insulator sites with decreasing H3K27me3 levels showed their relative enrichment from 5 to 30 kb distances from a Beaf32 site as shown (Fig. 2e). Unlike H3K27me3 spreading, H3K27me3 micro-domains localized in euchromatin, away from Beaf32 borders (Fig. 2f). Taken altogether, our data suggest that while the spreading of H3K27me3 levels occurs locally over Beaf32 borders, the concomitant decrease of H3K27me3 at distant micro-domains may involve long-range interactions with additional, distant GAF/dCTCF insulators.

### Micro-domain may form upon insulator-based long-range interactions

**Fig. 3:**
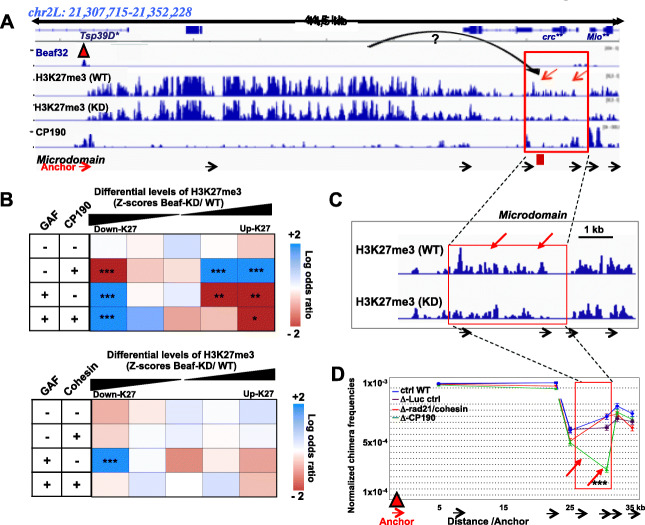
H3K27me3 micro-domains may be favored depending on long-range interactions with distant insulators depending on co-factors. **a** Genomic view of the Mio locus showing the ChIP-seq reads (*y*-axis) of Beaf32, H3K27me3, and CP190 in Beaf-KD-depleted and control cells. See panel **c** for a zoom of the micro-domain. The single asterisk indicates the downregulated genes (in Beaf-KD compared to WT control; e.g., Tsp39D) and double asterisks the upregulated genes (Mio (also called or “Mondo”) and crc). **b** Upper panel: Genome-wide analysis representing the relative enrichment (log odds ratio) of genes with decreasing (down-) or increasing (up-) H3K27me3 levels upon Beaf-KD compared to control cells, depending on binding of the indicated insulator protein co-factor CP190 alone or in combination with GAF (or dCTCF; not shown). Lower panel: same analysis for the insulator co-factor cohesin alone or in combination with GAF. The indicated *p* values (asterisks) were calculated using Fisher’s exact test in presence compared to absence of binding (see Additional file 3: Fig. S3). **c** Zoom of the micro-domain associated with the *Mio* locus of chromosome 2L. The arrows represent the sites where H3K27me3 levels decrease significantly upon Beaf-KD compared to control cells. **d** Chromosome conformation capture (3C) analysis of the long-range interactions with the micro-domain containing *Mio* promoter and a distant Beaf32 peak. The graph represents the relative frequency chimera products as measured by qPCR from Cohesin-depleted (blue), CP190-depleted (orange), or control siRNA-depleted (blue) cells. Proximal ligation products were estimated after HindIII restriction at the indicated Beaf32 (orange triangle) as anchor site using reverse primer and TaqMan-MGB probe (see “Methods”) with systematic measurements using primers spanning the whole *Mio* locus. Variations were tested by Student’s *t* test. (Additional file 3: Fig. S3C; reciprocal 3C)

Insulator proteins like dCTCF or Beaf32 contribute to the folding of chromosomes into TADs [26]. We hypothesized that weak TADs unable to restrain H3K27me3 within such topological unit might lead to micro-domain formation. An alternative possibility may involve the ability of insulator proteins to define specific LRIs with distant dCTCF or GAF [20, 50], independently of any contribution of insulators in assembling TADs. As an illustration, a H3K27me3 micro-domain was encountered at the distant GAF insulator sites flanking *Mio* locus, where Beaf32 establishes specific LRIs (Fig. 3a, c, red arrows) [50]. This micro-domain associated with *Mio* and *crc* genes was impaired upon Beaf32 depletion (Fig. 3a, c, red arrows). Beaf32 LRIs with GAF/dCTCF was shown to depend on co-factors including CP190 that is shared among all dCTCF, GAF, and Beaf32 types of insulators [50]. Accordingly, genome-wide analysis showed an enrichment of sites with most significant decreases in H3K27me3 levels in Beaf-KD cells when co-localizing with CP190, dCTCF, or GAF (Fig. 3b, upper matrix; *p* value of 1–4), in stark contrast to what was detected when it co-localizes with Beaf32 (Additional file 1: Fig. S3A-B). The involvement of CP190 was specific, contrasting with the additional co-factor cohesin that was not required for the decrease in H3K27me3 levels at GAF sites (Fig. 3b, lower matrix). Chromosome conformation capture (3C) further suggested that in contrast to CP190 depletion, depletion of cohesin did not affect long-range contacts at *Mio* as compared to control cells (Fig. 3d; Additional file 1: Fig. S3). Taken altogether, our data thus raised the possibility that H3K27me3 micro-domains form depending on presence of long-range interactions between insulator sites.

### Specific long-range contacts rather than TAD leakiness may account for micro-domains

**Fig. 4:**
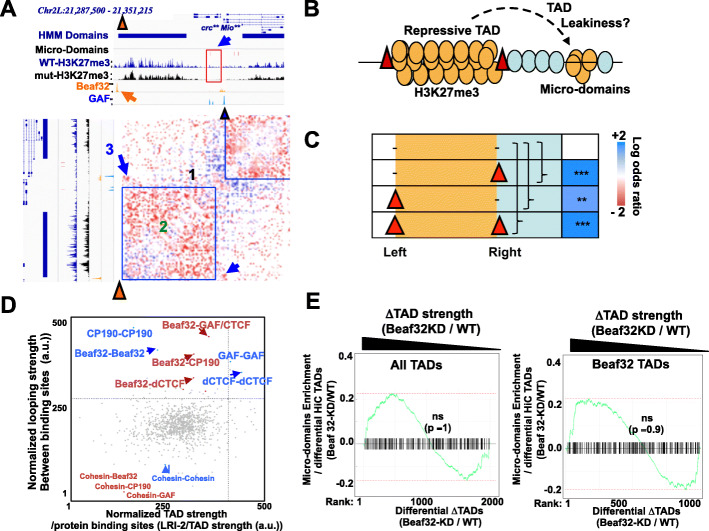
TAD insulation does not account for micro-domains. **a** Genomic view of *Mio* locus aligning the ChIP-seq data of H3K27me3 upon Beaf-KD and control cells identifying large heterochromatin domains by hidden Markov model (HMM) along with micro-domains (red rectangle), the ChIP-seq of Beaf32, and GAF insulator proteins and the 2D map of long-range interactions as obtained from sub-kb-resolution Hi-C data from S2 cells [21]. Beaf32 and GAF sites are represented by orange and blue triangles, respectively. Note that the *Mio* locus is representative of the enrichment of Beaf32 at TADs/compartments borders [26, 62]. **b** Scheme representing the genomic context of euchromatic micro-domains with respect to nearby Beaf32 sites near repressive heterochromatin TADs (see panel **c**), as detected by high-resolution Hi-C. **c** Enrichment test of micro-domains in euchromatin as a function of the presence or not of a particular arrangement of Beaf32 sites at borders of the heterochromatin domain. Colors represent odds ratio and asterisks the corresponding *p* values (by Fisher’s exact test), as calculated relatively to domains without any Beaf32 site (see accolades). Note that presence of Beaf32 sites on the left of the heterochromatin domain belong to loci enriched in micro-domains found on the opposite side of such domains (as illustrated by the *Mio* locus). **d** Scattered plot representing the scores obtained from genome-wide aggregated Hi-C assessing long-range interactions [9] with data from S2 cells [20], between the indicated binding sites of Beaf32, GAF, dCTCF, and their co-factors CP190 or cohesin. *X*-axis: aggregation of global long-range interactions estimating the strength of TADs (as normalized Hi-C reads in LRIs-2; see Fig. 5a) depending on the binding at their borders of the indicated insulator proteins versus control sites. *Y*-axis: estimate of strength of looping (as normalized Hi-C reads in LRIs-3; see Fig. 5a) between the indicated binding sites or control sites (gray dots). The vertical and horizontal lines represent the threshold of the top-5% of Hi-C interactions (of the total Hi-C bins). Note that similar results were obtained using various sources of Hi-C data (see Additional file 4: Fig. S4; see “Methods”). **e** Gene set enrichment analysis (GSEA) testing the influence of TAD strength on formation of H3K27me3 micro-domains. TAD strength was estimated by computing Hi-C data in Beaf32-depleted and control cells (see “Methods”). Differential TAD strength was measured as net variations between Hi-C data in Beaf32-depleted cells compared to control cells. GSEA was performed for all TADs (left) or among the TADs bordered by a Beaf32 site. Of note, this requires using the high-resolution 2000 TADs to reflect the genomic context of the test with repressive H3K27me3 TADs, as shown (Fig. 4b) (see “Methods”) [15, 20]. The normalized differential LRI scores were estimated for all loci defined by a couple of bins corresponding to one Beaf32 site interacting with any distant gene (> 5 kb) harboring a H3K27me3 micro-domain or not. Genes were also classified depending on additional parameters of aggregated Hi-C (see Fig. 5)

Our observations supported a model where Beaf32 regulates H3K27me3 micro-domains involving long-range interactions (Fig. 4a, b). In the case of *Mio*, the micro-domain was detected in the euchromatin domain localized on the opposite side of the Beaf32 site that flanks heterochromatin (Fig. 4a). Such an arrangement was found to be among the significant genomic contexts that favor micro-domains, providing the presence of a Beaf32 site on either side of heterochromatin (Fig. 4c; 336/722 micro-domains) or on both sides (4th row: 361/722 micro-domains).

**Fig. 5:**
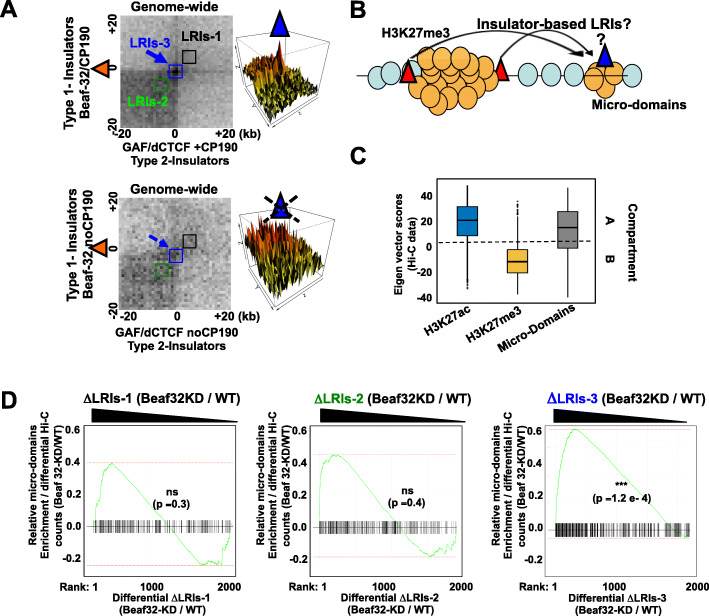
Aggregated Hi-C data highlight a role of insulator-based long-range contacts in micro-domains. **a** Aggregation of Hi-C data [9, 50] highlighting genome-wide long-range interactions between all Beaf32 sites (“Type-1 insulators”) with the distinct insulators bound by GAF or dCTCF (“Type-2 insulators”) depending on their co-localization with CP190 or not (upper and lower panels, respectively). LRIs 1, 2, and 3 represent A/B compartments (LRIs-1: long-range interactions detected between two A or two B domains), TADs (LRIs-2: long-range interactions defining TAD units in the Hi-C matrices), and specific loops (LRIs-3: long-range interactions between two defines sites (e.g., Beaf32, GAF, or dCTCF)), respectively, quantified for all TADs as normalized Hi-C reads (see “Methods”). Similar results were obtained when estimating LRIs from other Hi-C data [16, 20, 63] (Additional file 4: Fig. S4). **b** Scheme representing an alternative mode of 3D spreading of H3K27me3 into micro-domains; specific insulator-based long-range interactions between insulators may put them in physical proximity thereby favoring spreading from a repressive H3K27me3 domain to a micro-domain (see text). **c** Eigen value of micro-domains compared to control regions corresponding to active A compartments (e.g., domains harboring active genes and enriched in H3K27 acecylated histone marks) or inactive/repressive B compartments (enriched in H3K27me3 repressive histone marks) chromatin domains (see “Methods”). **d** Gene set enrichment analysis (GSEA) testing the influence of insulator-based variations in LRIs on formation of H3K27me3 micro-domains. ΔLRIs-1/2/3 were measured as net variations between Hi-C data in Beaf32-depleted cells compared to control cells (see “Methods”) [20, 21]. The normalized differential ΔLRI scores were estimated for all loci defined by a couple of bins corresponding to one Beaf32 site interacting with any distant gene (> 5 kb) harboring a H3K27me3 micro-domain or not. Genes were classified depending on differential ΔLRI-1/2/3 levels (left, middle and right graphs, respectively) to test which variations best predict the association of genes with a micro-domain (log *p* values were obtained using a corrected Fisher exact test)

Given the contribution of dCTCF or Beaf32 in TADs [26], our above observations raised the possibility that micro-domains form when TAD strength is low, i.e., when H3K27me3 sites in a repressive TAD may randomly spread onto the flanking euchromatin. In this instance, spreading into micro-domains might reflect TAD “leakiness” or weakness. In contrast, robust TADs might contribute to insulate euchromatin from flanking heterochromatin. We thus evaluated TAD strength using genome-wide aggregation analyses, as developed previously [9, 50] (see “Methods”) depending on protein binding. This analysis shows that Beaf32 binds to the borders of the most robust TADs genome-wide (Fig. 4d), which also involves GAF, dCTCF, and CP190 proteins. We then assessed the influence of Beaf32 depletion on all TADs genome-wide, testing if the probability to detect a micro-domain in the flanking euchromatin domain could be explained by the reduction in TAD strength, as tested using gene set enrichment analysis (GSEA) (Fig. 4e; see “Methods”). Ranking according to the changes in Hi-C counts representing TAD robustness (ΔLRI-2; see Fig. 5a) showed no significant correlation with the presence of micro-domains (*p* value =1 in both instances). As such, our results suggest that deregulation of TAD robustness by depletion of insulator proteins may not account for the presence of micro-domains.

Insulator binding sites not only bracket TADs, they also define sites with high levels of LRIs in the genome, as evidenced by aggregating Hi-C data onto their binding sites (Fig. 4d; Fig. 5a; see middle region (LRI-3) of the matrix). Such ability to form LRIs with distant sites is notably detected in presence of insulator proteins and cohesin or CP190 co-factors, reflecting how insulators are capable of forming long-range interactions (Fig. 5a, LRI-3). Of note, these are unique features specifically detected with insulator protein sites, and not found for control sites as shown by global assessment of LRIs as a function of protein binding (Fig. 4d, see *y*-axis). We thus reasoned that such loops between Beaf32 localized at the borders of repressive domains with distant sites (including GAF sites) inside euchromatin, may represent an alternative possibility accounting for H3K27me3 micro-domains (Fig. 5b). Inspection of the characteristic Eigen’s value reflecting euchromatin/heterochromatin into distinct A/B compartments (see “Methods”) showed that micro-domains may not be totally separated from B compartments (Fig. 5c). Thus, an alternative rationale for micro-domain formation may also be due to imperfect 3D compartmentalization of such euchromatic sites from heterochromatin.

To test these hypotheses in details, we first estimated the changes in long-range interactions upon Beaf32-depleted compared to control cells, reflecting either reduction in compartmentalization/phase separation (left: ΔLRIs-1) or alternatively in reducing specific loops (right: ΔLRIs-3) between insulator sites. We also compared such measures with possible changes in TAD robustness, as previously (middle: ΔLRIs-2). All TADs were then ranked according to the variations of each metric (Fig. 5d; ΔLRI) [20, 21], providing with three different genome-wide rankings of TADs. The influence of ΔLRI parameters was then tested using gene set enrichment analysis (GSEA) to assess which one best predicts the formation of micro-domains (Fig. 5d; see “Methods”). Ranking according to ΔLRIs between A compartments (LRI-1) or TAD strength (LRI-2) show no significant prediction of micro-domains. In stark contrast, ranking according to specific LRIs between Beaf32 and distant insulator (GAF/dCTCF) sites (ΔLRIs-3) show that ΔLRIs-3 significantly predicted micro-domain formation (*p* value = 1.2e−4). Accordingly, distant sites with LRIs not influenced by Beaf32 depletion showed lower chances to harbor micro-domains (Fig. 5d; compare left and right part of the curve). Therefore, specific long-range contacts (LRIs-3) define the best parameter accounting for micro-domain formation, as confirmed using various sources of Hi-C data (Additional file 1: Fig. S4)(see “Methods”). We conclude that the influence of insulator proteins on micro-domains more likely reflect their ability to establish specific long-range interactions rather than a global contribution to insulate domains or to assemble TADs.

### Beaf-KD impairs LRIs depending on CP190 at genome-wide levels

**Fig. 6:**
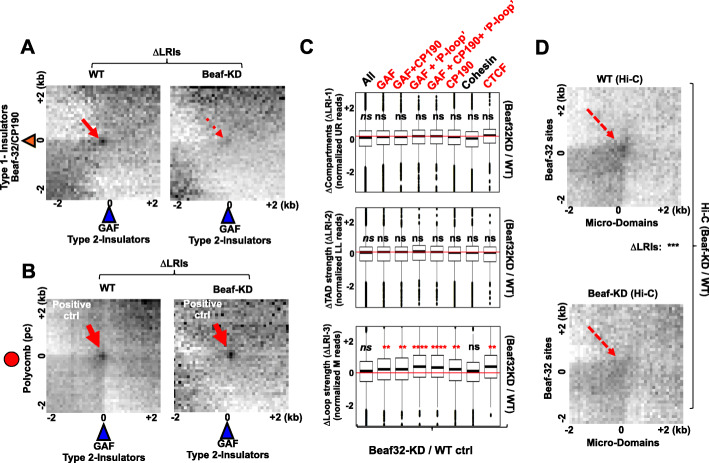
Hi-C data upon Beaf32 depletion confirms its role in specific long-range depending on presence of co-factors. **a** Aggregation of Hi-C data in control cells (right plot) and Beaf32-depleted cells (left plot) [20] highlighting genome-wide long-range interactions between all Beaf32 sites and GAF/dCTCF sites. The higher density of Hi-C reads in the middle region (red arrow) highlights specific looping (LRIs-3) between Beaf32 and GAF or dCTCF sites, which is reduced upon Beaf-KD (see panel **b** for a control). **b** Same as panel **a** except aggregation of Hi-C was performed between GAF and Pc/Polycomb (see “Methods”) [33]. Note that LRIs-3 were not significantly reduced in this instance. **c** Beaf32 depletion alters ΔLRIs between its binding sites and those of GAF or dCTCF, CP190, indirect peaks shown to predict loops (“P-loop”) with Beaf32 sites [50], dCTCF (lone) or cohesin, estimated as variations in LRIs-1 (compartments), LRIs-2 (TAD strength), or LRIs-3 (specific loops). LRIs-3 define the most significant parameter for detecting the impact of Beaf32 depletion (*p* values by the Wilcoxon pairwise test). **d** Genome-wide long-range interactions between distant Beaf32 sites and micro-domains as measured from Hi-C in control cells (upper panel) compared to Hi-C in Beaf32 depleted cells (Beaf-KD, lower APA panel). Note that LRIs were significantly reduced upon depletion (the *** indicates a significant *p* value <1e−4 as obtained by the Wilcoxon pairwise test)

Additional aggregation of Hi-C data highlighted loops/LRIs between Beaf32 and distant GAF/dCTCF/CP190 insulator sites in control cells, which were actually impaired upon Beaf32 depletion (Fig. 6a). In contrast, the loops formed between GAF sites and Polycomb/Pc were retained in depleted cells (Fig. 6b), confirming a specific influence. Most significant reductions in ΔLRI-3 were observed in presence of GAF, dCTCF, and CP190 binding indeed (Fig. 6c; Additional file 1: Fig. S5), whereas a systematic influence on LRIs assessing compartments or TAD strength could not be detected (Fig. 6c; ΔLRI-1 and ΔLRI-2, respectively). Beaf32 indirect peaks that predict loops [50] were enriched among the sites influenced for LRIs with distant Beaf32 sites upon Beaf32 depletion (Fig. 6c; Predicted “P-loop”). Importantly, micro-domains themselves formed significant LRIs with the distant Beaf32 sites, which were impaired by Beaf-KD (Fig. 6d). Therefore, our analyses show that Beaf32 is required for specific LRIs with distant insulators, which may account for the presence of H3K27me3 micro-domains.

### Synthetic insulator proteins impair both CP190 loading and H3K27me3 micro-domains

**Fig. 7:**
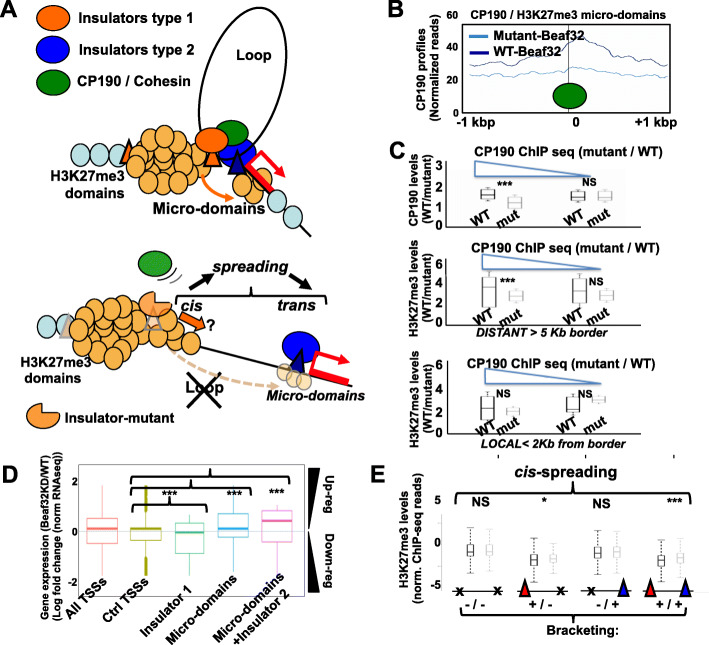
Insulator protein mutants impair H3K27me3 micro-domains depending on CP190 recruitment. **a** Upper: scheme representing the 3D-based formation of micro-domains involving the indicated molecular players of long-range interactions (LRIs-3; see panels **b**, **c**, **e**). Lower: scheme representing the impact of Beaf32 looping mutants on insulator-mediated LRIs by GAF /dCTCF and CP190 co-factors that results in both distant spreading onto micro-domains and (gain) in local spreading at borders. **b** Averaged H3K27me3 levels of previously identified micro-domains using normalized ChIP-seq data from cells expressing Beaf32 mutants compared to control cells (see “Methods”). **c** Upper panel: Box plot quantifying CP190 binding as normalized ChIP-seq reads in cells expressing Beaf32 mutants compared to control (see “Methods”) in two groups of micro-domains harboring decreasing CP190 levels (left) or not (right). Middle panel: Box plot showing the levels of H3K27me3 binding (normalized ChIP-seq reads) in Beaf32 mutants compared to control for the same micro-domains harboring decreasing CP190 levels or not (as defined in the upper panel). H3K27me3 levels were measured depending on proximity of micro-domains (< 1 kbp; lower panel) or not (> 5 kb; middle panel) to borders. **d** Box plot representing the differential gene expression analysis from RNAseq (as a log ratio of normalized RNAseq reads; see “Methods”) in Beaf32 mutants compared to control cells. A global negative influence of micro-domains was evidenced by a significant upregulation of the flanked genes (*p* value 1e−4; by the Wilcoxon pairwise test; for a total of 147 TSSs flanked by a micro-domain; see Tables S1-S2) as compared to control genes not flanked by a micro-domain or to genes near a Beaf32 site (“Insulator type 1”). Insulator-2 represents GAF or dCTCF. **e** Quantification of local spreading (at borders) depending on no bracketing (left), one-side bracketing by either Beaf32 (second box) or GAF/dCTCF CP190 (third box), and (as depicted in panel **a**) bracketing on both sides by Beaf32 and GAF/dCTCF CP190 (right; genomic context depicted in panel **a**). *p* values: Wilcoxon pairwise test for normalized levels in Beaf-KD/control cells (see Additional file 1: Fig. S6-S7)

We previously designed specific Beaf32 mutants that impaired looping due to their impaired ability to recruit CP190 onto insulator sites (Fig. 7a) [50], in complete agreement with the major role of CP190 in LRIs. We thus asked whether Beaf32 mutants could impair micro-domains due to failure to promote CP190-dependent looping with distant GAF/dCTCF insulators. Beaf32 mutants were expressed as previously [50], followed by ChIP-seq to score H3K27me3 variations systematically compared to control cells (see “Methods”). Enrichment tests showed that of the micro-domains identified in wild-type and that were lost in Beaf32-depleted cells, 55.5% (500/901) were also impaired by looping mutants (Additional file 1: Fig. S6A-E, *p* value of 1e−75), as confirmed by the reproducible decrease in H3K27me3 levels at micro-domains (Additional file 1: Fig. S6F). These results strongly supported the view that looping is a key feature required for micro-domain formation. GAF/dCTCF and CP190 binding sites were enriched in micro-domains harboring the most significant decreases in H3K27me3 levels in presence of mutants (Additional file 1: Fig. S6E, rows 1–2), supporting a central role of CP190 in micro-domain formation at distant GAF/dCTCF sites. Averaged CP190 profiles were decreased by the mutants (Fig. 7b) concomitantly with the decrease in H3K27me3 levels, for sites where CP190 was also decreased (Fig. 7c; upper and middle box plot, respectively). Of interest, the decreases in CP190 and H3K27me3 were most specific of micro-domains localized away (> 5 kb) from Beaf32 borders (Fig. 7c; middle box plot). In stark contrast, micro-domains flanking Beaf32 heterochromatin borders showed no decrease (Fig. 7c; Additional file 1: Fig. S6E, lower box plot), as such borders are subjected to H3K27me3 spreading locally (Fig. 1c), as confirmed by enrichment tests (Additional file 1: Fig. S6E).

Our work identifies micro-domains of H3K27me3 where heterochromatin components may “use” 3D loops to spread over distant sites (Fig. 7a). Such phenomenon was detected at hundreds of sites depending on specific long-range contacts with the insulator proteins GAF and dCTCF and their shared co-factors CP190 (Additional file 1: Fig. S6-S7), which is specifically impaired by expressing looping mutants as shown (Fig. 7a; lower scheme). Of interest, micro-domains contributed to control the expression of nearby genes that become upregulated upon depletion of Beaf32 (Fig. 7d; *p* value of 1e−4; see also Additional file 1: Fig. S6G). Such genes pertain to specific gene ontologies associated with distant spreading, such as the immune response, cellular homeostasis, and signal transduction (Additional file 1: Fig. S7), which are distinct from genes being regulated locally at Beaf32/dCTCF insulators [46, 64]. In the latter case, pairing of Beaf32 with GAF conditions the presence of micro-domains and it favors spreading locally (Fig. 7e; *p* value <1e−6). Hence, combinations of distinct insulators may be required to detect spreading across the heterochromatin borders. Thus, insulator bracketing may contribute to spreading in 3D, for micro-domain formation, and also for the demarcation of euchromatin from heterochromatin.

Taken altogether, our data support a functional implication of specific LRIs into gene expression programs. We propose that such LRIs contribute to regulate the spreading of H3K27me3 to distant sites, giving rise to micro-domains that participate to insulator-mediated homeostasis of gene expression throughout development (see “Discussion”).

## Discussion

Chromosome compartmentalization in 3D reinforces the demarcation of euchromatin from heterochromatin to control gene expression globally. The identification of micro-domains highlights that heterochromatin can further influence genes through specific long-range contacts in euchromatin. Micro-domain formation requires insulator-based LRIs between heterochromatin TAD borders and micro-domains, which does not contradict compartmentalization principles. The 3D organization of heterochromatin may therefore also influence expression through specific LRIs participating in H3K27me3 deposition locally, in micro-domains, thereby regulating distant euchromatic genes.

Compartmentalization principles may reinforce the global demarcation of TADs [57, 65]. Remarkably, recent high-resolution approaches in single cells have unraveled small “nano-compartments” that define TADs [1, 15]. Nano-compartments thus reflect how higher-order chromatin organization promotes interactions among domains sharing the same epigenetic state (A-A or B-B compartments) and self-interactions within the same folding TADs. Although H3K27me3 nano-compartments are self-maintainable, it remains unclear whether insulator factors, or transcription, participate to the demarcation of these domains from neighboring euchromatin. Our data highlight specific long-range contacts between the borders of nano-compartments with distant sites in euchromatin, through specific insulator-mediated loops. The resulting H3K27me3 micro-domains do not imply that TADs are not strong or that nano-compartments are ill-defined. Actually, LRIs between nano-compartments and nearby euchromatin are poor predictors of micro-domains. Rather, LRIs involved in micro-domain formation specifically involve TAD borders and they depend on insulator proteins. Therefore, micro-domains challenge classic models of insulator-based demarcation of H3K27me3. Rather, insulators do not solely “protect” nearby genes from spreading, as insulator-mediated looping also favors H3K27me3 spreading to distant sites in 3D.

Insulator proteins and additional factors participate to DNA looping between TAD borders [12, 20, 50], thereby contributing to the demarcation of epigenetic domains [30, 40, 65, 66]. Yet a structural role of insulators would predict that their removal alter heterochromatin-euchromatin barriers more systematically than what has been observed [59, 60]. Our work supports the view that the barrier activity of insulators further relies on combinations of insulator factors (Beaf32, GAF, and dCTCF or additional insulator proteins) (Fig. 7), in complete agreement with recent high-resolution Hi-C data [20, 55]. H3K27me3 spreading can actually occur through loops between two distant insulators [67], which may depend on insulator combinations and orientations [55]. Multiple insulators appear thus required for efficient H3K27me3 blocking at borders, while allowing spreading through 3D looping, depending on genomic contexts.

Pioneer work showed that CTCF participated in gene expression homeostasis [68], possibly due to CTCF/cohesion facilitating enhancer-promoter contacts inside TADs. Our data raise the possibility that a complementary contribution of insulators in expression homeostasis could involve loop-based H3K27me3 deposition. Actually, systematic detection of H3K27me3 throughout developmental stages of Drosophila embryos highlights high correlation coefficients in H3K27me3 levels among micro-domains compared to control euchromatin sites (Additional file 1: Fig. S7C-D). As LRIs are transient, persistence of H3K27me3 micro-domains through development may rely on Polycomb-encoded memory and histone-based positive feedback in 1D and in 3D [30, 69, 70]. Similar to Heterochromatin Protein 1-based liquid droplets [31] or to super-enhancers clustering [34], insulator-based micro-domains maintenance may depend on 3D clustering and phase separation principles [71]. Such clustering may serve to counteract high turnover dynamics by erasers/demethylases [40, 71–73]. A sub-fraction of micro-domains overlap with 9.7% of genomic enhancers (Additional file 1: Fig. S7E) that may also be regulated by Polycomb [74]. These observations suggest that co-regulation of H3K27me3 levels in micro-domains further involve shared transcriptional activators to subsets of enhancers.

Micro-domains are not unique in that previous observations identified dispersed, heterochromatin-like H3K9me2/3 islands, which may also depend on 3D organization [75]. Specific long-range interactions are involved in the nucleation of PRC2-mediated repression before allosteric spreading [76], which may involve CTCF-based assembly of TADs or looping [77]. Fly para-segment identity actually relies on specific LRIs at endogenous chromatin boundary insulators [54, 55]. Homeotic gene full repression requires Hox clustering through LRIs for full PRC2-dependent repression during development [78], even though repressive TADs may self-assemble [1]. Further studies should unravel how specific LRIs regulating H3K27me3 at distant genes, depending on dynamics of Pc clusters and co-factors binding at enhancers, TSSs, or insulators, could serve to progressively acquire gene expression homeostasis during development.

## Methods

### Cell culture, insulator mutants, RNAi, and gene expression analyses

Exponentially growing S2 cells were depleted by double-stranded RNAs (dsRNAs) against Beaf32, CP190, or cohesin (rad21) compared to mock-depletions (dsRNAs against luciferase) as previously described [50, 59], using the indicated oligos (see Additional file 4: Table S3). Gene expression analyses by RNAseq were performed as previously described [50] on cells depleted of Beaf32 or in cells expressing mutant or WT Beaf32 (GSE52887).

### Chromatin immunoprecipitation analyses and micro-domains detection

Chromatin immunoprecipitations were done as previously described [59] followed by high-throughput sequencing (ChIP-seq) with affinity-purified anti-CP190 antibodies [59] and anti-H3K27me3-specific antibodies (Upstate #07-449) performed in independent replicates in Beaf32-depleted cells and mock-depleted control cells, as well as in 2 × 2 cell replicates expressing mutant- or WT-Beaf32 (see “Methods” for details). For detection of micro-domains, we used all four ChIP-seq datasets analyzed as replicates of control cells compared Beaf32 depleted normalized to input, using normR package version 1.8.0, https://github.com/your-highness/normR developed by Helmuth and Chung for automated normalization and difference calling in ChIP-seq data [61], with the enrichR function using 40-bp bin sizes. Robustness of domain detection was tested according to various bin sizes (20 to 200 bp) and selection of domain sizes was performed for domains < 2 kb, based on variations (FDR < 5e−2) of the signal between depleted and control conditions (Additional Methods for details).

### 3C/Hi-C experimental and data analysis

All scripts used in this manuscript are available at: https://github.com/ CuvierLab/K27me3_mdom_spreading/tree/master/src. Hi-C data in both S2 cells and KC cells were normalized using K-R norm function Knight-Ruiz [9]. Aggregation analysis was performed as previously in 1D/2D/3D plots [9, 50, 79] using various sources of high-resolution Hi-C data [16, 20, 21] aggregated onto the H3K27me3 borders of repressive sub-TADs (of median size of 16 kb) depending on presence or absence of the indicated insulator proteins Beaf32, dCTCF, and GAF together with CP190 or cohesin binding by integrating previous ChIP-Seq data [45, 80]. Long-range interactions (LRIs) were estimated as previously described [9, 50] by extracting normalized intensities of the indicated LRIs at specific binding sites in Beaf-KD and control cells [20, 21] (see “Methods”). 3C measurements of LRIs in CP190-depleted, rad21-depleted, or control depletion (dsRNA against luc) conditions as performed by qPCR using TaqMan MGB probes as previously described [50]. Frequency of chimera was estimated in triplicates relatively to products from random ligation estimated using BACs that span the same loci (see Additional information for details).

## Supplementary information

**Additional file 1: Figure S1.** Genomic contexts of the influence of Beaf32 on H3K27me3 spreading. **Figure S2.** Validation of micro-domains by quantitative PCR analysis of ChIP. **Figure S3.** Regulation of H3K27me3 spreading by Beaf32 and CP190 and depending on genomic contexts. **Figure S4.** Regulation of long-range interactions by insulator proteins. **Figure S5.** Beaf32 depletion affects specific insulator-based LRIs by Beaf32, GAF, dCTCF and CP190. **Figure S6.** Beaf32 looping mutants alter H3K27me3 levels in micro-domains involving regulation of CP190 recruitment. **Figure S7.** Regulation of H3K27me3 trans-spreading may contribute to co-regulate specific gene functions through development.

**Additional file 2: Table S1.** List of genes associated with H3K27me3 micro-domains.

**Additional file 3: Table S2.** List of genes associated with the binding sites of insulator proteins.

**Additional file 4: Table S3.** List of oligos used in this study.

**Additional file 5.** Review history.

## Data Availability

All source codes pertaining to this manuscript are released in compliant with the Open source initiative (OSI) under MIT license and are accessible in GitHub https://github.com/CuvierLab/H3K27me3_micro-Dom_spreading [81] and the Zenodo doi: https://zenodo.org/record/3889838#.Xut4gpMza_u [82]. All ChIP-seq data pertaining to this manuscript were deposited to GEO of NCBI (GSE130211) [83], RNAseq are accessible through GSE52887 [50]. The corresponding lists of H3K27me3 micro-domains are provided in Tables S1-S2, alone or in association with nearby genes, respectively (see Additional information).
