# Supplementary material for: Insulator-based loops mediate the spreading of H3K27me3 over distant micro-domains repressing euchromatin genes
Source: Genome Biol. 2020 Aug 3;21:193. doi: 10.1186/s13059-020-02106-z (PMC7397589; doi:10.1186/s13059-020-02106-z)
Supplement: Supplementary file 1 — Additional file 1: Figure S1. Genomic contexts of the influence of Beaf32 on H3K27me3 spreading. Figure S2. Validation of micro-domains by quantitative PCR analysis of ChIP. Figure S3. Regulation of H3K27me3 spreading by Beaf32 and CP190 and depending on genomic contexts. Figure S4. Regulation of long-range interactions by insulator proteins. Figure S5. Beaf32 depletion affects specific insulator-based LRIs by Beaf32, GAF, dCTCF and CP190. Figure S6. Beaf32 looping mutants alter H3K27me3 levels in micro-domains involving regulation of CP190 recruitment. Figure S7. Regulation of H3K27me3 trans-spreading may contribute to co-regulate specific gene functions through development. [file 13059_2020_2106_MOESM1_ESM.pdf]

SUPPLEMENTARY MATERIALS

Insulator-based loops mediate the spreading of H3K27me3 over distant micro-domains repressing euchromatin genes

Alexandre Heurteau[+], Charlène Perrois[+], David Dépierre[+], Olivier Fosseprez[+], Jonathan Humbert[+^], Stéphane Schaak[+] and Olivier Cuvier[+@]

ADDITIONAL METHODS

Cell culture, siRNA depletions

Drosophila S2 cells were grown in Schneider Drosophila medium (Invitrogen) supplemented with 10% Fetal Bovine Serum (FBS) and 1% antibiotic (streptomycin; GIBCO). For dsRNA treatment, S2 cells were diluted to 10 x10^6 cells/ml 1 day before treatment and then diluted at 1 x10^6 cells/ml in media without serum for dsRNA treatment for 2.5 h at 25°C before adding back FBS and harvested after 4 days followed by RNA extraction (for RT-qPCR). Depletions were performed using double stranded RNAs (dsRNA) specific for beaf32, cp190, cohesin/rad21 subunit or mock dsRNA (luciferase) as previously done (1) and using the oligos listed (see **Table S3**), after checking for potential off-target effects using NCBI primer designing tool and dsCheck. Preparation of dsRNAs, was done as previously (1, 2), using the indicated oligos (see **Table S3**) for T7-driven transcription (Fermentas TranscriptAidTM T7 High Yield Transcription Kit). Gene expression was measured by quantitative RT-qPCR analysis using cDNAs prepared from S2 control, Beaf32- CP190 or Cohesin/rad21 -depleted cells, for the indicated oligos (**see Table S3**). Levels of mRNAs were quantified by RTqPCR

with >3 different concentrations of cDNA for standard curves with Biorad iQ SYBR Green Supermix in Applied biosystems viia7 (ThermoFisher). For synthetic mutant- and WT-Beaf32, we used previously characterized stably transfected cell lines expressing either form of synthetic Beaf32 from previously stably transfected S2 cell lines (2), as obtained upon stable integration of pUWG-neo vectors selected by neomycin resistance. Of note, these cells harbor highly reduced levels of endogenous *beaf-32* expression as compared with synthetic mutant- or WT- Beaf-32 as shown (2).

Characterization of H3K27me3 micro-domains

For analyses of H3K27me3 levels, Reads were first trimmed with bbduk.sh script from BBtools (38.00) and with standard parameters. The alignments were performed with Bowtie2 (2.3.3.1) on dm3 genome version with -q10 parameter. Samtools rmdup (1.4.1) was then used on sorted BAM files to filter out potential PCR duplicates. Bigwig files were generated with deeptools BamCoverage (3.0.1-2-2) RPKM normalized and using a bin size of 50bp with a smoothing window of 150 bp. H3K27me3 micro-domain detection by NormR was performed in two steps. First, detection was performed for normalized ChIP-seq read counts compared to input. Second, a systematic comparison was performed as normalized ChIP-seq read counts comparing Beaf32 depleted (or mutant Beaf32) to control (**Figure 2**; see also **Additional file 2 and 6: Fig. S2** and **S6**). The preferential distribution of micro-domains as nucleosome 'mers' was verified by taking various bin sizes for detection (10, 20 and 40 bp) and not detected from control domains as obtained after running NormR on control input samples (e.g. input 1/2/3 compared to input 2/3/1) as systematically quantified in comparison with micro-domains. Further selection was performed using FDR of 5x10-2 (see NormR). Micro-domains were further selected based on sizes (< 2 kb) (**Additional file 2: Fig. S2D**) and after excluding those micro-domains nearby (< 2kb) heterochromatin borders (to avoid mixed

signals due to local spreading; see Figure 1). Micro-domains were further tested by quantification of H3K27me3 in mutants or upon Beaf32 depletion compared with WT (**Additional file 6: Fig. S6**). For quantification of H3K27me3 levels inside micro-domains, we quantified normalized ChIP-seq read counts by comparing systematically the first two quartiles between Beaf32 depleted and control cells, as well as between synthetic mutant/WT Beaf32 expressing cells. H3K27me3 micro-domains were analyzed by counting the ChIP-seq reads for H3K27me3 normalized over input levels of bins corresponding to micro-domains compared to heterochromatin and euchromatin bins (bins from heterochromatin and euchromatin domains). Heterochromatin H3K23me3 domains were identified as large (> 5 kb) blocks of heterochromatin by Hidden Markov Model (HMM)(R script) hence defining heterochromatin domain borders (Figure 1) as previously (1). Further intersection analyses of micro-domains showing significant reduction were then performed directly using genomic coordinates of domains with enhancers (STARR-seq) (3, 4) or the binding sites of GAF (5), dCTCF (6) and Beaf32 (2), or in the case of differential expression analyses with RNAseq, after filtering micro-domains associated with (+/-1 kb) with TSSs. For quantification of insulator protein binding near micro-domains, we used normalized ChIP-seq reads in windows (+/-1 kb) surrounding micro-domains compared to levels found at random euchromatic sites (same number of sites taken randomly from the same euchromatin domains). For the analysis with respect to heterochromatin borders, error bars represent the variations between replicates for all bins in the indicated intervals. Normalized H3K27me3 levels were measured for all bins and plotted to compare the H3K27me3 distribution in heterochromatin, euchromatin or micro-domains bins (**Additional file 2: Fig. S2**). To validate the genome-wide impact of Beaf-KD compared to control cells, on variations of H3K27me3 (Figure 1E), we chose to score H3K27me3 in +/-1 kb windows surrounding transcription start sites as we previously showed that 91.1% of Beaf32 sites mapped in this context (2).

Experimental and computational integration of 3C and Hi-C data

TADs were inferred using HiCseg algorithm after normalizing 1kb contact matrices as previously performed (7) using Kc167 Hi-C data from Li *et al*. (8) allowing us to monitor long-range interactions within such TADs (696 contiguous TADs of a median size ~110Kbp). All interactions were measured using Hi-C data from S2 and Kc167 cells and assessed within the limits of the calculated TAD domains, with one exception for GSEA test (see below). Extraction of long-range contacts was performed using dump command from juicer tools in order to obtain normalized contacts (observed/expected and Knight-Ruiz correction). 2D APA plots were generated with heatmap function from R stats library after importing dumped matrices. as previously described (9). Aggregation first was performed by aggregating long-range contacts onto the indicated binding sites in 1D (2) and then in 2D/3D essentially as previously described by 'Aggregate Peak Analysis' (APA)(9) run onto the normalized Hi-C data from S2 or KC cells (10, 11). LRIs were measured from the aggregated matrices to probe specific loops at the binding sites (LRIs-3; 3x3 squares at middle of plot), or in the lower left part of the 2D plot (LRIs-2; 3x3 squares reflecting TAD strength normalized to 3x3 squares on lower right) or compartments (LRIs-1; upper right (3x3 squares) normalized to lower right). For statistical tests probing LRIs variations between Beaf32-KD and control cells (12), we used Fisher exact test after genome-wide ranking of TADs depending on LRI 1/2/3s. Further estimation was done by estimating variations of LRI1/2/3s between Beaf-32 depleted and control cells at the indicated binding sites, using pair-wise Wilcoxon tests. LRIs 1-2-3 levels were calculated by taking the means of normalized interactions that fall in a 3x3 contiguous non overlapping squares as represented in Figure 4C. To optimize statistical ranking test by Gene set enrichment analysis (GSEA), we utilized high-resolution Hi-C detecting approximately 2,000 TADs throughout the genome (12), which correspond to

repressive TAD domains (Figure 4B). A/B compartments were detected with the eigenvector function from juicer tools (v 0.7.5) with parameters KR and at 10 kb resolution. To retrieve a homogeneous A/B nomenclature for chromosomes 2L 2R 3L 3R and X, we further verified that arbitrary A compartment labeling was associated with active genes as detected by RNAseq (from **GSE22069**). Then, compartment eigenvector were estimated in parallel and for bins of similar sizes corresponding to either A- or B- compartments, or to the detected micro-domains. A/B compartments were reconstructed as previously described (13). Briefly, a positive or negative score was attributed to the eigen values depending on the first type of compartment encountered in the PCA (see /PROCESSING_SCRIPTS/COMPARTMENT _COMPUTATION/ under linked Github repository(14) https://github.com/CuvierLab/ H3K27me3_micro-Dom_spreading and the zenodo doi: https://zenodo.org/record/3889838#. Xut4ppMza_v) (15), for every Hi-C bin and by defining compartments by presence of active genes (13). After identifying A/B compartments, we inverted the sign of eigen vector so every A (or B) compartment had positive (or negative) value, respectively. For 3C data, quantification of LRIs was further estimated as chimera religation products in 3C samples depleted of CP190-, rad21 Beaf32 or control (luciferase) depleted cells as measured by qPCR using MGB-Taqman probes as previously (2) with the same probes. Briefly, 60 millions of cells were collected after short crosslinks (0.8% PFA; 10 min) followed by addition of 125 mM glycine and 3x PBS washes. Digestion with concentrated 2000u Hind III (NEB R0104M) was performed after neutralization of 0.2% SDS with Triton X100 (1.2% final), at 37°C overnight and then with another 500u for 2 hours followed by a stop reaction (1% SDS at 65°C for 20 min) before adding T4 ligation buffer (1/20 ; v/v) for a 4h incubation at 16C with T4 DNA ligase (200u; Fermentas) and 1 mM ATP. EDTA (1M) was added and DNA recovered after Phe/chloroform extraction and ethanol precipitation. DNA products were measured by qPCR (Viia7 ; Life Technology) with bona fide reverse primers (PCR

efficiencies of 95-100 %). 3C chimeras were normalized to random ligation using the same conditions from DNA purified from BACs spanning the same regions (BACR34H23 and BACR05D08) with Taqman-MGB (16). Standard deviation was calculated for each condition from independent replicates measured in triplicates as previously done (2).

Epigenetic domains during development

H3K27me3 ChIP-seq data along all Drosophila developmental stages were obtained from modencode (17, 18). Correlation coefficients were calculated through a K-S test and a Pearson correlation value for each micro-domain compared with control bins of the same size distribution. Control bins were selected out of euchromatin regions (filtering out bins from heterochromatin and domain borders) as for micro-domains.

Expression analyses

RNA-seq analysis was performed as previously (1) with the RNAseq data from Beaf32-depleted compared to siRNA mock depleted (luciferase) control cells (GSE57168). Boxplot for gene expression analysis was generated using log ratio of BeafKD/WT as normalized RNA-seq reads by taking the normalized DEseq2 output counts.

ADDITIONAL TABLES

Table S1. List of genes associated or not with euchromatic H3K27me3 micro-domains in presence or absence of the indicated insulator proteins (see Material and Methods for details). The columns correspond to: the micro-domain number, the chromosome name, the start of the micro-domain, the end of the micro-domain, the distance to nearest border (of a H3K27me3 domain), the distance to nearest euchromatin border (non-H3K27me3 domain), log fold change of H3K27me3 signal (Beaf32-KD/ WT), zscore of H3K27me3 (Beaf32-KD/ WT), normalized differences of H3K27me3 signal (Beaf32-KD/ WT), log fold change of H3K27me3 signal (Beaf32-KD/ WT), zscore of H3K27me3 signal (Beaf32-KD/ WT), differences of H3K27me3 signal (Beaf32-KD/ WT).

Table S2. List of genes associated with H3K27me3 micro-domains and with the binding sites of insulator proteins dCTCF, GAF/M1BP, Beaf-32, and their shared co-factors CP190 or cohesin. Note that only CP190 is enriched in insulator-based micro-domains that disappear upon depletion of Beaf-32 (see text). The columns correspond to: the micro-domain number, the chromosome name, the start of the micro-domain, the end of the micro-domain, the width (in number of unit), the strand (+ or -), the FBgn gene id (Dm6 release), the presence of micro-domain associated with the gene (<1 kb), the presence or not of a binding site for - Beaf32, dCTCF, CP190, GAF, Cohesin, DREF, the up-regulation of genes (upon Beaf-KD compared to WT control), the FBgn and Dm6 gene name (Flybase).

Table S3. List of oligos used for generating dsRNAs-mediated depletion of cohesin, CP190 or Beaf-32 (see Material and Methods), oligos used for RTqPCR analyses validating depletions or oligos for ChIP-qPCR analyses of H3K27me3 levels verifying the presence or absence of micro-domains in control or depleted depletions.

ADDITIONAL REFERENCES

1. Lhoumaud P, Hennion M, Gamot A, Cuddapah S, Queille S, Liang J, et al. Insulators recruit histone methyltransferase dMes4 to regulate chromatin of flanking genes. EMBO J. 2014;33:1599-613.

2. Liang J, Lacroix L, Gamot A, Cuddapah S, Queille S, Lhoumaud P, et al. Chromatin immunoprecipitation indirect peaks highlight long-range interactions of insulator proteins and Pol II pausing. Mol Cell. 2014;53(4):672-81.

3. Arnold CD, Gerlach D, Stelzer C, Boryn LM, Rath M, Stark A. Genome-wide quantitative enhancer activity maps identified by STARR-seq. Science. 2013;339(6123):1074-7.

4. Zabidi MA, Arnold CD, Schernhuber K, Pagani M, Rath M, Frank O, et al. Enhancer-core-promoter specificity separates developmental and housekeeping gene regulation. Nature. 2015;518(7540):556-9.

5. Gilchrist DA, Nechaev S, Lee C, Ghosh SK, Collins JB, Li L, et al. NELF-mediated stalling of Pol II can enhance gene expression by blocking promoter-proximal nucleosome assembly. Genes Dev. 2008;22(14):1921-33.

6. Negre N, Brown CD, Shah PK, Kheradpour P, Morrison CA, Henikoff JG, et al. A comprehensive map of insulator elements for the Drosophila genome. PLoS Genet. 2010;6(1):e1000814.

7. Mourad R, Cuvier O. Computational Identification of Genomic Features That Influence 3D Chromatin Domain Formation. PLoS Comput Biol. 2016;12(5):e1004908.

8. Li L, Lyu X, Hou C, Takenaka N, Nguyen HQ, Ong CT, et al. Widespread rearrangement of 3D chromatin organization underlies polycomb-mediated stress-induced silencing. Mol Cell. 2015;58(2):216-31.

9. Rao SS, Huntley MH, Durand NC, Stamenova EK, Bochkov ID, Robinson JT, et al. A 3D map of the human genome at kilobase resolution reveals principles of chromatin looping. Cell. 2014;159(7):1665-80.

10. Rowley MJ, Nichols MH, Lyu X, Ando-Kuri M, Rivera ISM, Hermetz K, et al. Evolutionarily Conserved Principles Predict 3D Chromatin Organization. Mol Cell. 2017;67(5):837-52 e7.

11. Eagen KP. Principles of Chromosome Architecture Revealed by Hi-C. Trends Biochem Sci. 2018;43(6):469-78.

12. Wang Q, Sun Q, Czajkowsky DM, Shao Z. Sub-kb Hi-C in D. melanogaster reveals conserved characteristics of TADs between insect and mammalian cells. Nat Commun. 2018;9(1):188.

13. Fortin J-P, Hansen KD. Reconstructing A/B compartments as revealed by Hi-C using long-range correlations in
epigenetic data. Genome Biol. 2015;this issue.

14. Heurteau A, Perrois C, Depierre D, Fosseprez O, Humbert J, Schaak S, et al. Insulator-based loops mediate the spreading of H3K27me3 over distant micro-domains repressing euchromatin genes. Github. 2020;https://github.com/CuvierLab/H3K27me3_micro-Dom_spreading.

15. Heurteau A, Perrois C, Depierre D, Fosseprez O, Humbert J, Schaak S, et al. Insulator-based loops mediate the spreading of H3K27me3 over distant micro-domains repressing euchromatin genes. Zenodo. 2020;https://zenodo.org/record/3889838 - .XuvFkZMza_s.

16. Court F, Miro J, Braem C, Lelay-Taha MN, Brisebarre A, Atger F, et al. Modulated contact frequencies at gene-rich loci support a statistical helix model for mammalian chromatin organization. Genome Biol. 2011;12(5):R42.

17. Roy S, Ernst J, Kharchenko PV, Kheradpour P, Negre N, Eaton ML, et al. Identification of functional elements and regulatory circuits by Drosophila modENCODE. Science. 2010;330(6012):1787-97.

18. Kharchenko PV, Alekseyenko AA, Schwartz YB, Minoda A, Riddle NC, Ernst J, et al. Comprehensive analysis of the chromatin landscape in Drosophila melanogaster. Nature. 2011;471(7339):480-5.

19. Rowley MJ, Corces VG. Minute-Made Data Analysis: Tools for Rapid Interrogation of Hi-C Contacts. Mol Cell. 2016;64(1):9-11.

20. Ramirez F, Bhardwaj V, Arrigoni L, Lam KC, Gruning BA, Villaveces J, et al. High-resolution TADs reveal DNA sequences underlying genome organization in flies. Nat Commun. 2018;9(1):189.

21. Emberly E, Blattes R, Schuettengruber B, Hennion M, Jiang N, Hart CM, et al. BEAF regulates cell-cycle genes through the controlled deposition of H3K9 methylation marks into its conserved dual-core binding sites. PLoS Biol. 2008;6(12):2896-910.

ADDITIONAL FILES: FIGURES S1-S7

Fig S1. **Genomic contexts of the influence of Beaf32 on H3K27me3 spreading**

A. Box plot quantifying the levels of H3K27me3 at borders of heterochromatin domains in Beaf32 depleted ('Beaf32-KD') compared to control cells ('WT control'). The p-value was estimated using a Wilcoxon pair-wise test (see Materials and Methods).

B. Genome-wide analysis showing the enrichment (Log odds ratio) for down- and up-regulated genes compared to control (not deregulated) genes as measured by differential expression analysis after RNAseq in Beaf32-depleted compared to control cells (see Materials and Methods) according to gene ranked by differential H3K27me3 levels (Beaf32-depleted /WT control cells). Note that only down-regulated genes were uniquely enriched among genes harboring increasing H3K27me3 levels in contrast to up-regulated genes (p-values calculated by Fisher exact test are indicated by asterisks).

C. Scheme representing the genomic context tested in panel B depending on presence or not of Beaf32 sites on left, right or both sides of the domain. H3K27me3 levels were estimated in the 2 kb windows flanking borders (arrow in orange).

D. Box plot quantifying the extent of H3K27me3 spreading on the flanking euchromatin domain (first 2 kb on the left side of the domain), depending on the bracketing of domains by +/- Beaf32 sites on either side, both sides or no bracketing controls. Note that similar increases in H3K27me3 levels were observed whether domains harbor two Beaf32 sites or one Beaf32 site on the left side (p-value < 1e-4) compared to domains with no site or with one Beaf32 on the other side of the euchromatin domain (p-value of 1 and 1e-1, respectively)(see also **Figure 1 and 7**).

E. Volcano plot showing the differential expression of genes upon depletion of Beaf32 compared to control cells. X-axis: log fold change (LogFC); y-axis: Log p-value (Log-pval). Note that Beaf32 depletion did not significantly deregulate the Drosophila esc, Su(z)12 and E(z) encoding the subunits of PRC2 complex, as indicated .

F. Genome-wide analysis representing the relative enrichments of sites associated with H3K27me3 variations scored depending on the presence or not of the indicated insulator protein sites (Beaf32, Cohesin, GAF, dCTCF, DREF). 1 kb genomic bins were systematically ranked according to variations in H3K27me3 levels between Beaf32-KD and control cells (see Additional Methods). All sites (bound or not) were ranked in quintiles according to Log odds ratio (Beaf-KD/WT) and enrichment tests were performed the ranked sites are enriched in insulator protein binding sites. The indicated p-values (asterisks) were calculated using a Fisher's exact test. Note that the test was performed independently of TSSs.

Fig S2. **Validation of micro-domains by quantitative PCR analysis of ChIP**

A. Box plot of H3K27me3 levels quantifying normalized ChIP-seq reads in micro-domains in Beaf32-KD as compared to WT control cells (***; p-value < 1e-4; as tested by Wilcoxon pairwise test)(see **Additional file 2: Fig. S2** for the corresponding averaged plots).

B. Graph showing the qPCR analysis of H3K27me3 levels normalized to inputs in Beaf32-KD compared to control cells, for 16 micro-domains detected by ChIP-seq compared with control loci (see also panel C). Note that the 16 micro-domains also harbor significant loss of H3K27me3 upon Beaf32 mutants (see **Figure 6**).

C. Box plot summarizing the qPCR data validating decreasing normalized H3K27me3 levels in Beaf32-KD compared to control cells. The significant p-value (p= 2e-5) was calculated by pair-wise Wilcoxon test.

D. Box plot of H3K27me3 levels quantifying normalized ChIP-seq reads in micro-domains depending on their sizes, for Beaf32-KD compared to control cells (***; p-value < 1e-4; as tested by Wilcoxon pairwise test).

E. Same as in D except that micro-domains were split into two groups (> or < 2 kb) corresponding to the threshold used to select micro-domain (p-value was calculated by Wilcoxon pairwise test).

Fig S3. **Regulation of H3K27me3 spreading by Beaf32 and CP190 and depending on genomic contexts**

A. Genome-wide analysis showing the enrichment (log odds ratio) of genes flanked by the indicated insulator protein co-factor CP190 alone or in combination with Beaf32, depending on gene ranking by their decreasing or increasing H3K27me3 levels in Beaf32-KD / control cells (log p-values were calculated by a Fisher exact test; indicated by asterisks).

B. Same as in panel A except the combination of Beaf32 sites was analyzed depending on co-localization with cohesin binding sites.

C. Quantification by RTqPCR of the expression of cp190 and cohesin/rad21 genes upon RNAi treatment compared to control cells. Mock control dsRNAs (against luciferase ('luc2')) or dsRNAs specific of cp190 or of the rad21 subunit of cohesin were used to impair the expression of the corresponding genes as previously described (2). Following cDNA preparation, expression of these (cp190, cohesin/rad21) or of control genes (actin, gapdh) were then measured by qPCR in triplicates with normalization to standard curves (see Materials and Methods). Note that Beaf32 expression is globally unchanged in cp190 as compared to cohesin depleted cells, thereby arguing against possible indirect influence of cp190 on LRIs due to beaf32 expression.

D. Chromosome conformation capture (3C) analysis of long-range interactions between the *mio* gene –associated micro-domain and the distant Beaf32 peak near the TSS of *tsp39D* ('tsp39D')(see also **Figure 3A**). The graph represents the relative frequency chimera products as measured by qPCR from CP190-depleted (green) compared to control siRNA-depleted (black) cells (see also **Figure 3**). Proximal ligation products were estimated after HindIII restriction with reciprocal anchor primer flanking the micro-domain to probe long-range contacts with the Hind III fragments spanning the *tsp39* locus, using TaqMan-MGB probe (see Additional Methods). Variations were tested by Student's t-test in triplicates.

Fig S4. **Regulation of long-range interactions by insulator proteins**

Genome-wide aggregation of long-range interactions (2, 9) using high-resolution Hi-C data from KC cells (11) similar to what was detected in S2 cells (see **Figure 4**) (19, 20). Aggregation of Hi-C data in KC cells confirm genome-wide long-range interactions between all genomic Beaf32 sites with GAF or dCTCF sites depending on their co-localizing with CP190 (left) or not (right). 1, 2 and 3 represent A/B compartments (LRI-1: long-range interactions detected between two A or two B domains), TAD (LRI-2: long-range interactions defining triangles in the Hi-C matrices (as detected in panel B) and specific loops (LRI-3: specific long-range interactions between two defines sites (e.g. Beaf32, GAF or dCTCF), respectively. Note that similar results were obtained when estimating LRIs from other sources of Hi-C data (12, 19).

Fig S5. **Beaf32 depletion affects specific insulator-based LRIs by Beaf32, GAF, dCTCF and CP190**

Intersection matrix testing the influence of Beaf32 on LRIs-1/2/3 using Hi-C from Beaf32 depleted cells compared to control S2 cells (see Materials and Methods) (12). The net

variations of LRIs were measured for the 3 types of long-range interactions (LRI-1/2/3; see **Additional file 4: Fig. S4**), lost of LRIs between A-compartments (delta-LRI-1; left), lost of LRIs inside TADs (or TAD strength) (delta-LRI-2; middle graph) or lost of LRIs at insulator sites (LRI-3; right graph). Differential delta-LRIs-1/2/3 gene scores were then tested for enrichments depending on binding of the indicated insulator proteins (log p-values were obtained using a Fisher exact test).

Fig S6. **Beaf32 looping mutants alter H3K27me3 levels in micro-domains involving regulation of CP190 recruitment**

A. Venn diagram showing the intersection between H3K27me3 micro-domains lost in Beaf32-depleted cells and H3K27me3 micro-domains loss upon expression of synthetic Beaf32 mutants compared to WT Beaf32. P-value was calculated using Fisher exact test.

B. Right: Plot showing the genome-wide averaged H3K27me3 levels in previously identified micro-domains for cells expressing synthetic Beaf32 mutants compared to control cells expressing synthetic Beaf32 wild-type ('WT') using previously described stable cell lines (2)(see Material and Methods). The p-value was calculated using a wilcoxon pair-wise test. Left: Box plot showing the variations of H3K27me3 levels in micro-domains for cells expressing synthetic Beaf32 mutants compared to control cells expressing synthetic Beaf32 wild-type ('WT'). The p-value was calculated using a wilcoxon pair-wise test.

C. Venn diagram showing the intersection between H3K27me3 micro-domains independently identified in replicates of WT Beaf32 compared to synthetic Beaf32 mutants. P-value was calculated using Fisher exact test.

D. Quantification of spreading in *trans* scoring the variations in H3K27me3 levels of micro-domains depending on expression or not of Beaf32 mutants that are unable to recruit CP190 as shown (2). Variations were scored systematically depending on association with insulator

sites Beaf32, GAF, dCTCF, CP190 and indirect peaks reflecting long-range interactions with Beaf32 sites (2). Note that Beaf32 and CP190 sites are associated with increasing H3K27me3 levels supporting their role in cis-spreading (**Figure 1**).

E. Box plots showing the binding of Beaf32, GAF and CP190 (as normalized ChIP-seq reads; left-, middle- and right- box plot, respectively) next to (+/-1 kbp) micro-domains or to control sites, taken randomly from the same euchromatin domains context. The p-values were calculated by Wilcoxon tests (see Additional Methods).

F. The left box plot shows the normalized levels of H3K27me3 in two independent replicates of cells expressing either Beaf32 wild-type or mutants ('WT1' and 'WT2' and 'mut1' and 'mut2'). The H3K27me3 levels were measured for the same micro-domains identified in one set of replicate (WT2/mut2) to test the reproducibility in detecting higher H3K27me3 in the second replicate (WT1/mut1) as validated by comparing the signal in wild-type versus mutant condition. The right box plot shows the same analysis at micro-domains or at control sites (see Additional Methods). The p-value was calculated using a Wilcoxon pair-wise test.

G. Plot showing the positioning of gene TSSs relatively to the center of micro-domains (x-axis; position 0). Genes correspond to up-regulated and control genes (red and blue bars, respectively), as detected in cells expressing mutant compared to wild-type Beaf32 (see Additional Methods). Note that up-regulated genes are relatively enriched close to micro-domains (see also **Additional file 7: Fig. S7**). P-value calculated using a Fisher exact test.

Fig S7. **Regulation of H3K27me3 trans-spreading may contribute to co-regulate specific gene functions through development**

A. Intersection matrix testing the probability of formation of micro-domains (spreading in *trans*) in flanking insulator sites including GAF, dCTCF and CP190, depending on micro-

domains lengths (nucleosome mers; see also **Figure 2**). Note the enrichment of 'indirect' peaks that reflect the long-range contact sites of Beaf32 (2).

B. Micro-domains influence specific gene functions including regulation of immune response and of cellular homeostasis. Note that these GOs are uniquely associated with genes flanking micro-domains (unlike insulator proteins). GOs associated with insulator-based regulations in *cis* control distinct functions including the cell cycle and apoptosis as previously shown (21).

C. Assessing global correlation of H3K27me3 levels in euchromatin sites throughout development. Correlation statistics were performed through all developmental stages to assess correlation of H3K27me3 levels for sites corresponding to micro-domains and compared with control sites in the same environment (euchromatic sites, > 2 kb away from heterochromatin). ChIP-seq of H3K27me3 at all stages of development were from modEncode (GSE15292; see Materials and Methods) (17, 18).

D. Box plot showing the global correlation of H3K27me3 levels for euchromatin sites corresponding to micro-domains compared with euchromatic sites of the same sizes and in the same environment (see Materials and Methods). P-value was obtained by Wilcoxon test.

E. Venn diagram showing the intersection between micro-domains and +/-1 kb regions centered over the middle of annotated enhancers (4)(see Materials and Methods). P-values were calculated by a Fisher exact test. Note that P-value is not significant for intersections with shorter enhancer intervals (+/- 0.5 kb surrounding the annotated enhancers).

Fig S1

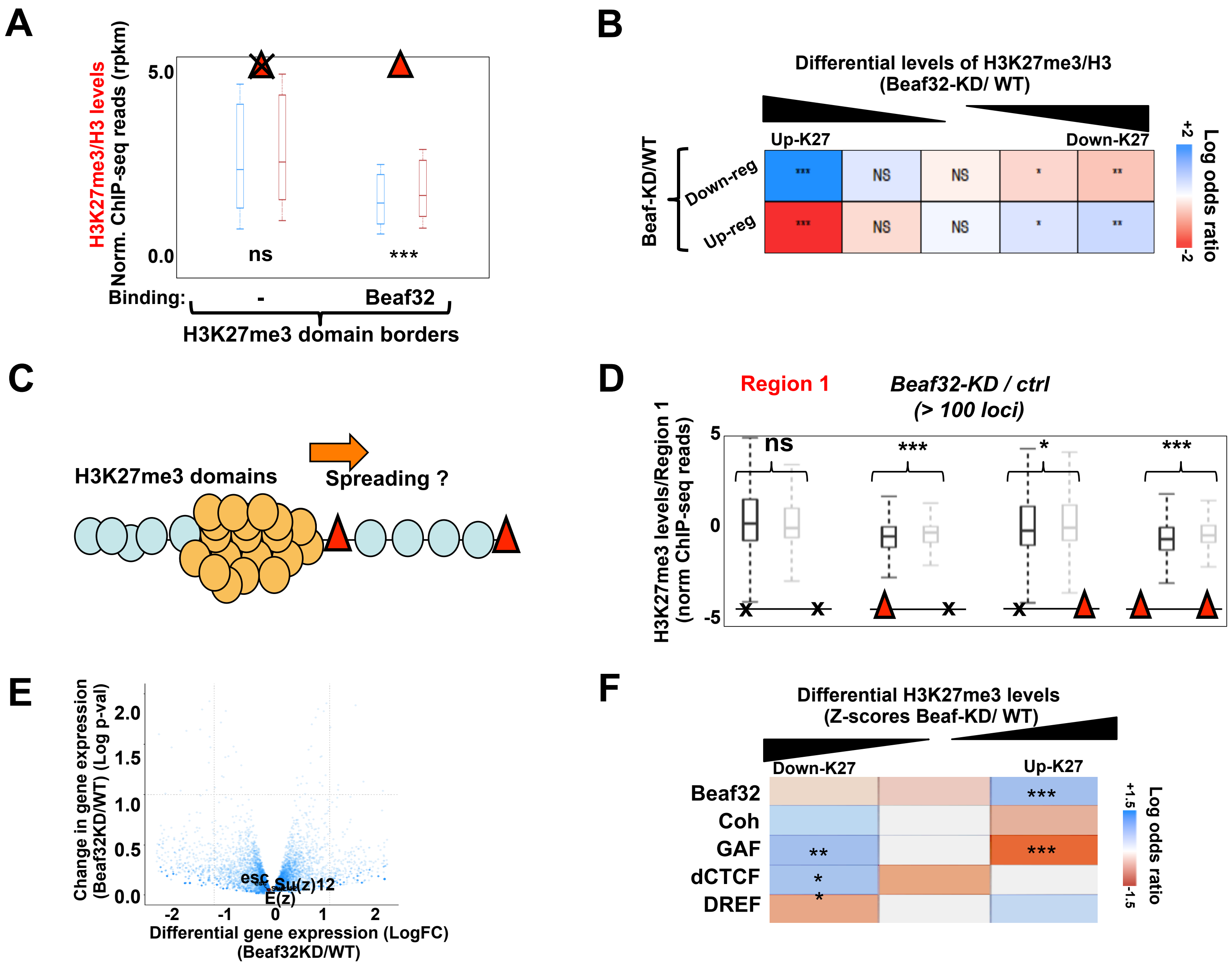
A
H3K27me3/H3 levels
Norm. ChIP-seq reads (rpkm)
5.0
0.0
ns
***
Binding:
-
Beaf32
H3K27me3 domain borders
B
Differential levels of H3K27me3/H3
(Beaf32-KD/ WT)
Up-K27
Down-K27
Beaf-KD/WT
Down-reg
Up-reg
NS
Log odds ratio
+2
-2
C
H3K27me3 domains
Spreading ?
D
Region 1
Beaf32-KD / ctrl
(> 100 loci)
H3K27me3 levels/Region 1
(norm ChIP-seq reads)
5
0
-5
ns
***
*
***
E
Change in gene expression
(Beaf32KD/WT) (Log p-val)
2.0
1.5
1.0
0.5
0.0
esc
Su(z)12
E(z)
-2
-1
0
1
2
Differential gene expression (LogFC)
(Beaf32KD/WT)
F
Differential H3K27me3 levels
(Z-scores Beaf-KD/ WT)
Down-K27
Up-K27
Beaf32
Coh
GAF
dCTCF
DREF
***
**
*
Log odds ratio
+1.5
-1.5

Fig S2

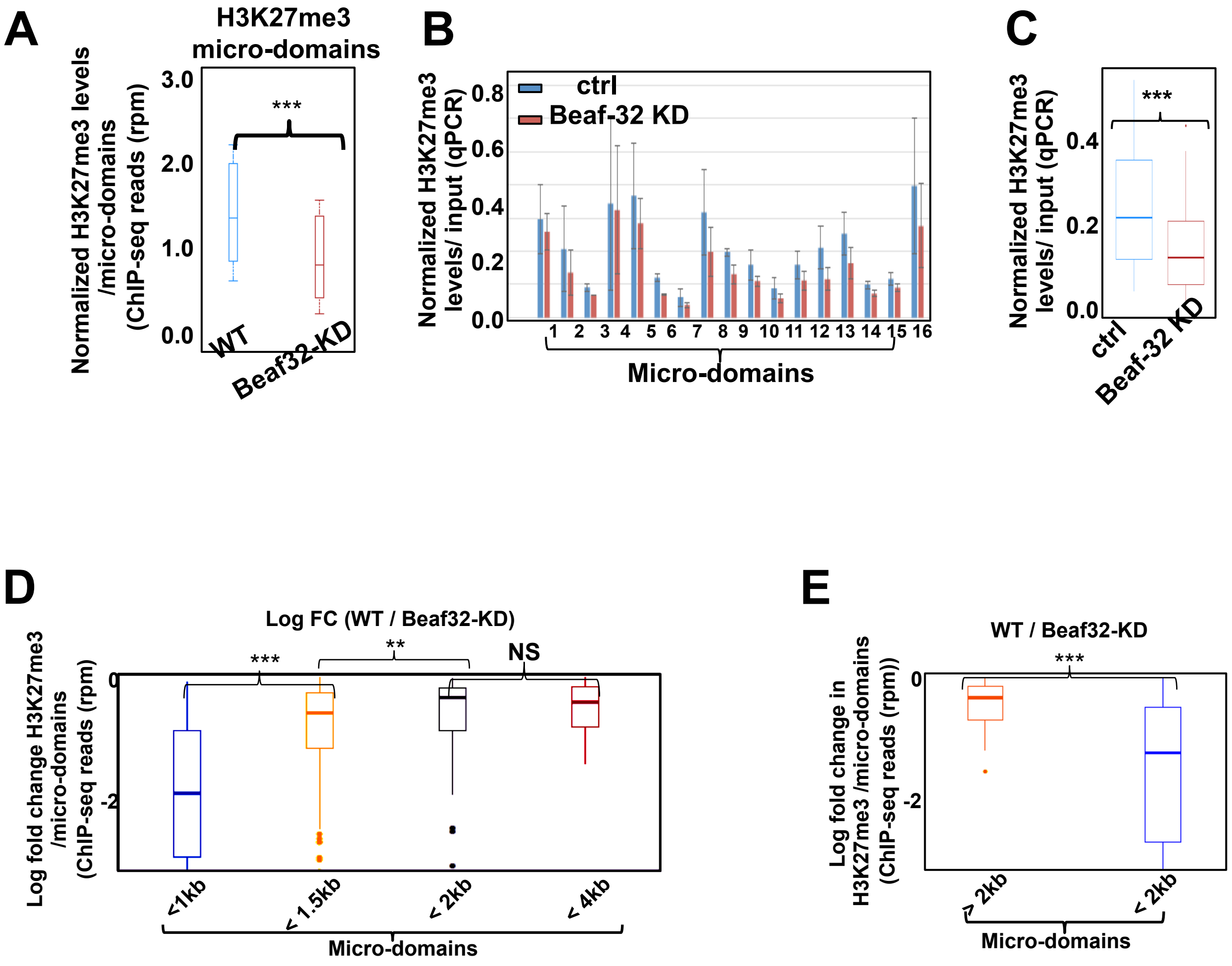
A
H3K27me3 micro-domains
Normalized H3K27me3 levels /micro-domains (ChIP-seq reads (rpm))
3.0
2.0
1.0
0.0
***
WT
Beaf32-KD
B
Normalized H3K27me3 levels/ input (qPCR)
0.8
0.6
0.4
0.2
0.0
ctrl
Beaf-32 KD
1 2 3 4 5 6 7 8 9 10 11 12 13 14 15 16
Micro-domains
C
Normalized H3K27me3 levels/ input (qPCR)
0.4
0.2
0.0
***
ctrl
Beaf-32 KD
D
Log FC (WT / Beaf32-KD)
Log fold change H3K27me3 /micro-domains (ChIP-seq reads (rpm))
0
-2
***
**
NS
<1kb
< 1.5kb
< 2kb
< 4kb
Micro-domains
E
WT / Beaf32-KD
Log fold change in H3K27me3 /micro-domains (ChIP-seq reads (rpm))
0
-2
***
> 2kb
< 2kb
Micro-domains

Fig S3

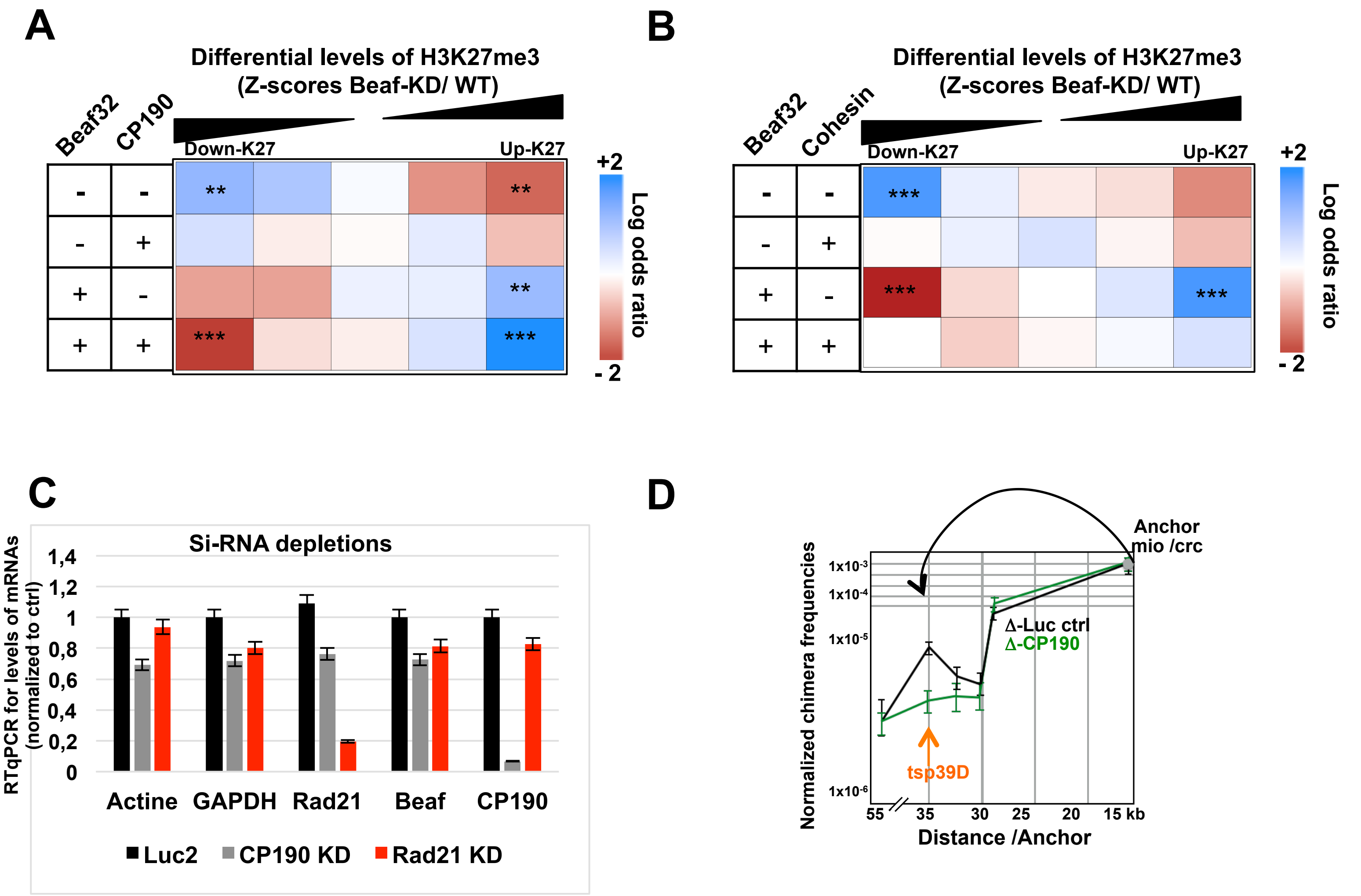
A
Differential levels of H3K27me3
(Z-scores Beaf-KD/ WT)
Beaf32
CP190
Down-K27
Up-K27
**
**
**
***
***
+2
- 2
Log odds ratio
B
Differential levels of H3K27me3
(Z-scores Beaf-KD/ WT)
Beaf32
Cohesin
Down-K27
Up-K27
***
***
***
+2
- 2
Log odds ratio
C
Si-RNA depletions
RTqPCR for levels of mRNAs
(normalized to ctrl)
1,4
1,2
1
0,8
0,6
0,4
0,2
0
Actine
GAPDH
Rad21
Beaf
CP190
Luc2
CP190 KD
Rad21 KD
D
Anchor
mio /crc
Normalized chimera frequencies
Δ-Luc ctrl
Δ-CP190
tsp39D
55
35
30
25
20
15 kb
Distance /Anchor

Fig S4

A

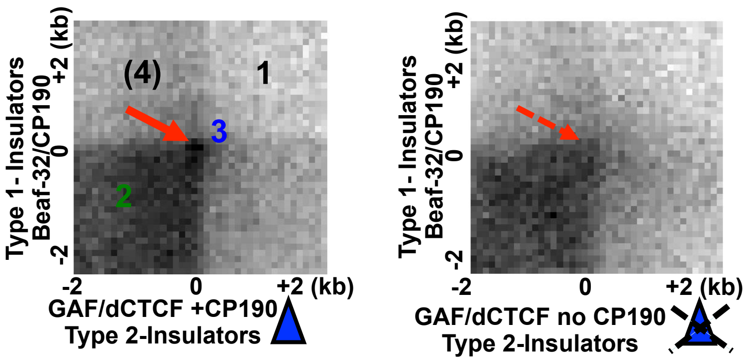

(4)
1
3
2
Type 1- Insulators
Beaf-32/CP190
+2 (kb)
0
-2
-2
0
+2 (kb)
GAF/dCTCF +CP190
Type 2-Insulators
Type 1- Insulators
Beaf-32/CP190
+2 (kb)
0
-2
-2
0
+2 (kb)
GAF/dCTCF no CP190
Type 2-Insulators

Fig S5

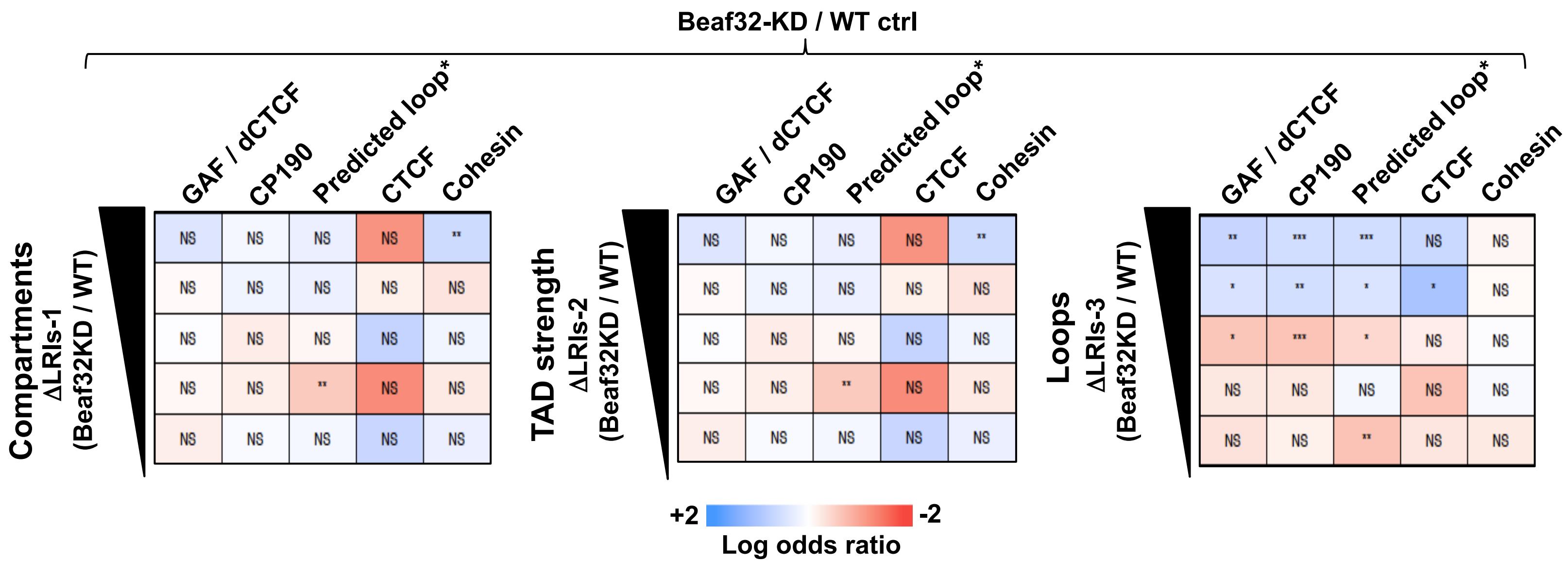
Beaf32-KD / WT ctrl
GAF / dCTCF
CP190
Predicted loop*
CTCF
Cohesin
Compartments
ΔLRIs-1
(Beaf32KD / WT)
TAD strength
ΔLRIs-2
(Beaf32KD / WT)
Loops
ΔLRIs-3
(Beaf32KD / WT)
+2
-2
Log odds ratio

Fig S6

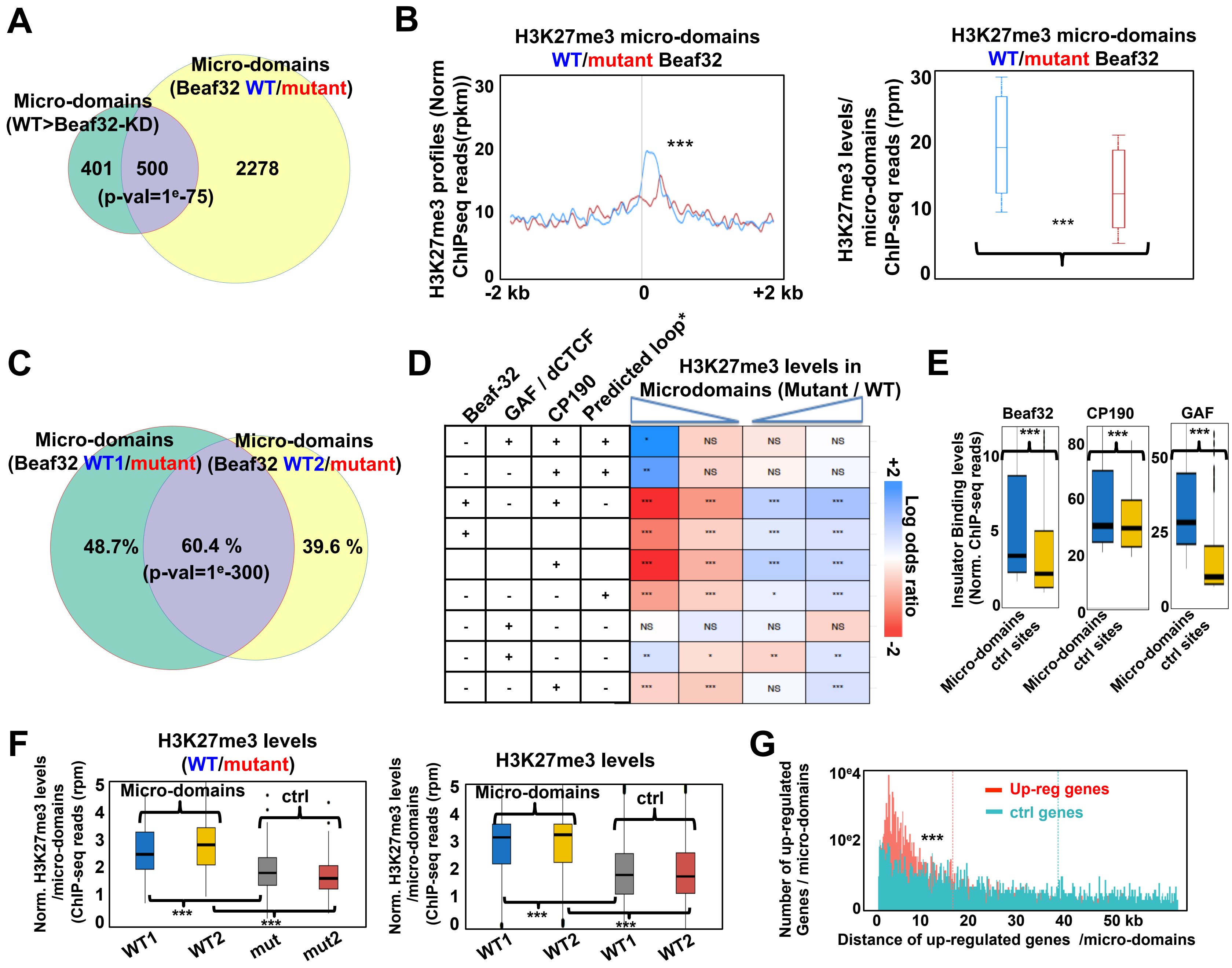
A
Micro-domains (Beaf32 WT/mutant)
Micro-domains (WT>Beaf32-KD)
401
500
2278
(p-val=1e-75)
B
H3K27me3 micro-domains WT/mutant Beaf32
H3K27me3 profiles (Norm ChIPseq reads(rpkm))
***
-2 kb
0
+2 kb
H3K27me3 levels/ micro-domains ChIP-seq reads (rpm)
C
Micro-domains (Beaf32 WT1/mutant)
Micro-domains (Beaf32 WT2/mutant)
48.7%
60.4 %
39.6 %
(p-val=1e-300)
D
Beaf-32
GAF / dCTCF
CP190
Predicted loop*
H3K27me3 levels in Microdomains (Mutant / WT)
Log odds ratio
+2
-2
E
Beaf32
CP190
GAF
Insulator Binding levels (Norm. ChIP-seq reads)
Micro-domains
ctrl sites
F
H3K27me3 levels (WT/mutant)
Norm. H3K27me3 levels /micro-domains (ChIP-seq reads (rpm))
Micro-domains
ctrl
WT1
WT2
mut
mut2
H3K27me3 levels
G
Number of up-regulated Genes / micro-domains
Up-reg genes
ctrl genes
Distance of up-regulated genes /micro-domains
50 kb

Fig S7

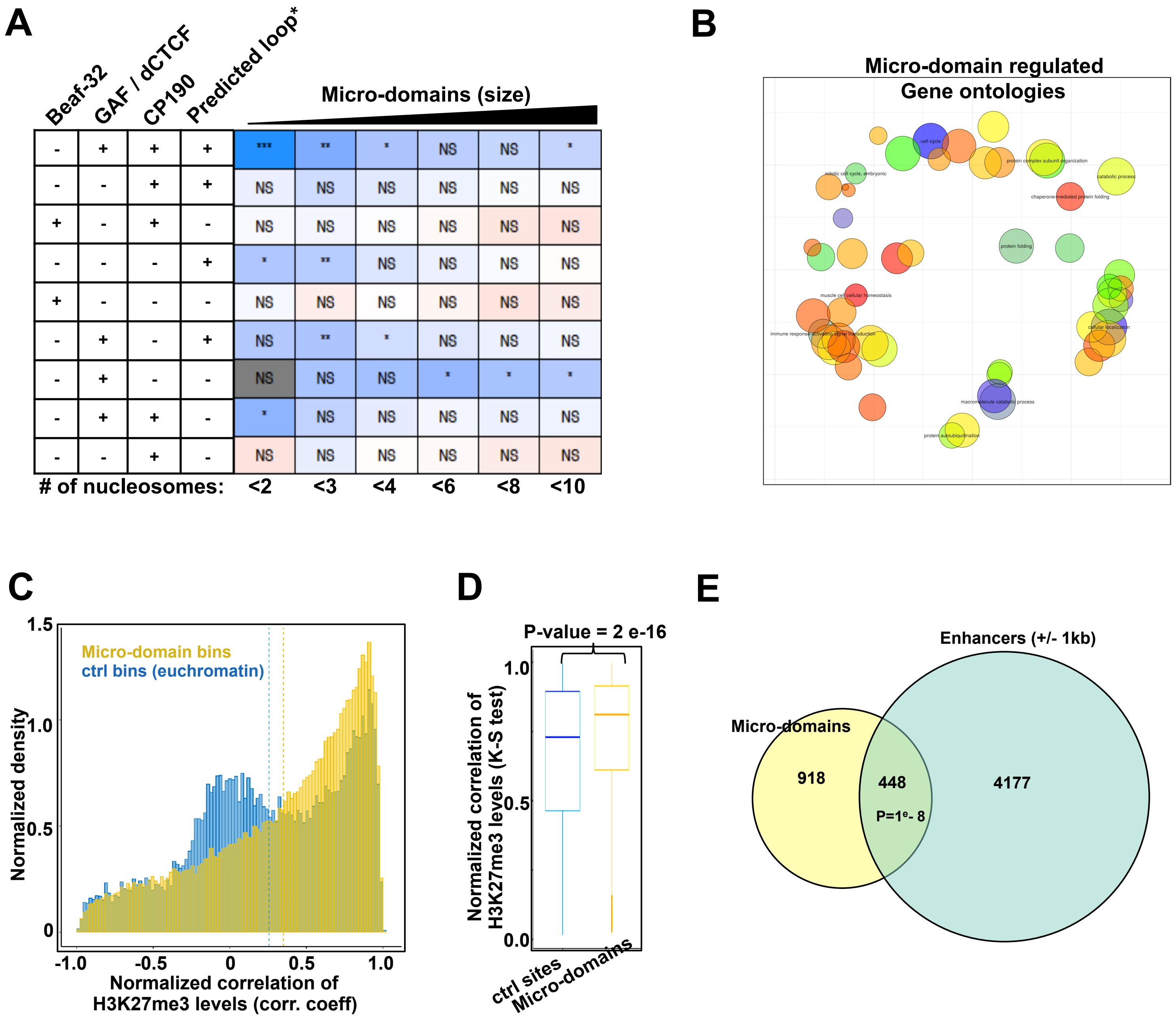
A
Beaf-32
GAF / dCTCF
CP190
Predicted loop*
Micro-domains (size)
- + + + *** ** * NS NS *
- - + + NS NS NS NS NS NS
+ - + - NS NS NS NS NS NS
- - - + * ** NS NS NS NS
+ - - - NS NS NS NS NS NS
- + - + NS ** * NS NS NS
- + - - NS NS NS * * *
- + + - * NS NS NS NS NS
- - + - NS NS NS NS NS NS
# of nucleosomes: <2 <3 <4 <6 <8 <10
B
Micro-domain regulated
Gene ontologies
C
Micro-domain bins
ctrl bins (euchromatin)
Normalized density
1.5
1.0
0.5
0
-1.0
-0.5
0
0.5
1.0
Normalized correlation of
H3K27me3 levels (corr. coeff)
D
P-value = 2 e-16
Normalized correlation of
H3K27me3 levels (K-S test)
1.0
0.5
0.0
ctrl sites
Micro-domains
E
Enhancers (+/- 1kb)
Micro-domains
918
448
P=1e- 8
4177
