# Supplementary material for: Insulator-based loops mediate the spreading of H3K27me3 over distant micro-domains repressing euchromatin genes
Source: Genome Biol. 2020 Aug 3;21:193. doi: 10.1186/s13059-020-02106-z (PMC7397589; doi:10.1186/s13059-020-02106-z)
Supplement: Supplementary file 5 — Additional file 5. Review history. [file 13059_2020_2106_MOESM5_ESM.docx]

Review History

**First round of review**

**Reviewer 1**

**Are you able to assess all statistics in the manuscript, including the appropriateness of statistical tests used?**

No, I do not feel adequately qualified to assess the statistics.

**Comments to author:**

This article is focused on the identification and characterization of the H3K27me3 micro-domains that result from long-distance interactions between regulatory elements. The authors suggest that long-distance contacts between regulatory elements form loops that bring H3K27me3 repressive regions into a close vicinity of the target genes. In other words, repressors like PREs can form large repressive domains in cis and at the same time function over the long distances similar to enhancers in trans. It was previously shown that interactions between insulators form a loop that brings in close proximity Polycomb Responsible Element (PRE) and the promoter of a reporter gene. As a result, PRE facilitates binding of Pc proteins (formation of a microdomain) to the promoter and induces its repression. Here, the authors analyzed the spreading of H3K27me3 marks depending on the presence or absence of the BEAF protein in the Drosophila cells. They found that BEAF is required for the boundary function genome-wide. At the same time, inactivation of BEAF decreases H3K27me3 levels at microdomains bound by the GAF, CP190, and dCTCF proteins. In general, the demonstration of the BEAF participation in boundary function and the finding of H3K27 microdomains are interesting. At the same time, the article is written in such a way that sometimes it is difficult to follow the presentation of results and many conclusions require additional explanations and more detailed descriptions of the experiments to justify the conclusions made by the authors. Therefore, it would be necessary to significantly revise the presentation of the results, which would allow a better assessment of their significance.

Additional comments:

1. In Figures 1D and 2E, it is not clear what is actually shown by the horizontal brackets, and why brackets for the BEAF-32 positive and negative borders are overlapping.

2. I would like to see the reproducibility of the H3K27me3 micro-domains between the ChIP-seq replicates.

3. It is very hard to follow Figure 3D. Please, show positions of the anchor and test fragments at the genomic diagram (similar to the panel A).

4. Significant changes of the contact frequencies measured in 3C should be validated in the reciprocal experiments. Authors should place an anchor primer at the restriction fragment demonstrating a decrease in the interaction frequency with the mio promoter upon the CP190 depletion, and measure its interaction frequency with several test fragments, including the mio promoter.

5. To state that BEAF-32 KD reduces H3K27me3 level at the studied micro-domain via the disruption of LRI, authors should perform 3C experiments with the anchor primers at the BEAF-32 binding site and micro-domain in the BEAF-32-deficient cells.

**Reviewer 2**

**Are you able to assess all statistics in the manuscript, including the appropriateness of statistical tests used?**

No, I do not feel adequately qualified to assess the statistics.

**Comments to author:**

How the 3D organisation of the genome may contribute to gene regulation is a key question of major current interest. The discovery that genomes are organized into topologically-associated domains (TADs) has focused attention on the role of intra-domain interactions. Here the authors propose a role for interactions between domains and distant sites. They propose that domains rich in the repressive histone mark H3K27me3 are involved in interaction with specific distant sites. This is proposed to be mediated by interaction between the insulator protein Beaf-32, flanking the H3K27me3 domains, and other insulator proteins located at the distant sites. This interaction would, through loop formation, bring the distant site into proximity with the HK27me3 domain leading to accumulation of H3K27me3 at the distant site and the down regulation of nearby genes. This idea of a function for insulators to recruit specific sites within nearby euchromatic domains to repressive domain "hubs" leading to regulation of chromatin state at the distant sites is an interesting proposal of broad interest to others in the field.

The evidence presented includes 1) the identification of over 1,000 micro-domains (<2kb) characterized by decreased levels of H3K27me3 in Beaf-32 knock-down versus wild type; 2) the significant enrichment of insulator proteins GAF and CP190 in micro-domains (Fig 3B); the significant association of micro-domains with sites of interaction between Beaf-32/Cp190 and GAF/dCTCF+CP190 that are sensitive to loss of Beaf-32 (Fig 5D) and the upregulation of genes neighbouring micro-domains upon Beaf-32 knock-down (Fig 7D).

However, evaluation of the above evidence is complicated as the paper is difficult to read and there are several issues with presentation of the data which is, in places, inconsistent and confusing.

Specific issues

1. The abstract is difficult to read and needs simplifying to concentrate on the main message of the paper; e.g. a) The first sentence introduces TADs and sub-TADS, then the second sentence talks about the association of "small sub-TADs" with insulators: what does this say about large sub-TADs and TADs? The paper seems to be about Beaf-32 flanking TADs rather than sub-TADs or even small sub-TADs. b) it is suggested that mechanisms preventing the rather undefined concept of compartmentalization "leakage" need to be explored but then it turns out that this is not what the paper is about at all.

2) Micro-domains are identified on the basis of reduced H3K27me3 in Beaf-32 knockdown so the reduction shown in Fig 2C is not surprising, but why the negative Fold Change in Fig2 B is only seen for micro-domains smaller than 2Kb is unclear.

3) Fig 3 indicates two micro-domains in a specific genomic region but, unhelpfully, they are indicated vaguely by arrows rather than definitively by extent. This is made more confusing in Fig 4 where only one micro-domain is indicated at this position. This is important as it affects the interpretation of the 3C result in Fig3D which shows an effect on interaction to the right side of the box but little effect at the interaction point on the left side of the box, which seems to be the closest point to the micro-domain.

4) the evidence presented in favour of the model is largely aggregate genomic data that need support from specific instances where clear effects can be observed; e.g. in the chosen selected region in Fig 3 there seems to be no evidence for a specific local interaction between the Beaf-32 site and the micro-domain. It would also be important to show specific local changes in gene expression associated with micro-domains in a number of selected genome regions.

Overall, although the authors present data to support their hypothesis, an alternative is that the effects on H3K27me3 at micro-domains are secondary effects to Beaf-32 knock-down. It will always be difficult to completely rule out secondary effects, but clear examples of specific gene regions with interactions and effects on gene expression supporting their proposal are required for the case for their hypothesis to be compelling.

More detailed issues

1) The title on the manuscript is problematic as there is no evidence that the micro-domains flank euchromatic genes. Generally the use of "flank " in the manuscript is potentially confusing as it is not clear whether it means "occurs on one side of" or "occurs on both sides of". Curiously a different title is given in the cover letter; this title seems better although it's not clear what the domains are distant from.

2) nomenclature of "trans-spreading". The spreading from large H3K27me3 domains to micro domains appears to be distant spreading in cis rather than "trans". "Distant spreading" would distinguish it from the local spreading at borders.

3) p3 line 19 "Such chromosomal…" this sentence is confusing as it combines two separate phenomena.

4) p5 line 34 "Such defects are not systematic…" what defects?

5) p5 line 54 "While depletion.." this sentence is confusing as it suggests that depletion of insulator proteins leads to spreading of H3K27me3 into micro-domains; the opposite of what is observed.

6) p6 line 19 What is the evidence that it is finely-tuned?

7) p7 Heading. "are associated with" might be better than "flank", which is rather confusing.

8) p8 line 22 "sites harbored relatively higher H3K27me3 levels" is confusing as line 44 says the micro-domains had "low H3K27me3 levels".

9)p9 line 12 "Micro-domains are distinct from…heterochromatin domains" presumably some H3K27me3 peaks that are reduced in Beaf-32 knock-down overlap with large H3K27me3 domains. What, if any, is this overlap?

10) p9 "epigenetically stable"; is the stability of microdomains addressed?

11) p10 line 17 not clear what "genes" means here; are only sites overlapping transcription units being analysed? Needs clarification/justification.

12) p10 line 53 "mio" gene. This is confusing as the coordinates given do not fit with the mio gene located at 2L:2,217,290..2,220,625 according to Flybase (rel6 coordinates). Also the coordinates given in Fig 3 span 44.5kb whereas the Figure indicates 40kb. Additionally the coordinates given in Fig 3 do not make sense with the coordinates given in Figure 4; the right edge of Fig 3 is given as 21,352,228 but Fig4, which apparently extends further to the right, is given as 21,351,215.

13) p11 line 2 why focus the analysis arbitrarily on the 722 micro-domains <1kb from TSS? Needs justification.

14) Fig 1. What do the dotted boxes represent? The ones over micro-domains are confusing as they have different dimensions to the indicated microdomains. What micro domains are indicated; the 1,311 list or the 722 list? Do the red bars just indicate micro-domain position or do they represent domain lengths? Blue triangle "on top of genes"; meaning unclear. Is there a significance to the length of brackets in 1E? Why does 1E focus on genes (i.e. transcription units) and how are marks allocated to genes?

15) Fig 2B. It's not clear to me how micro-domains can have lengths that are smaller than the resolution of ChIP peaks.

16) Fig 3D The sample colours in the Figure do not correspond to the description in the Figure legend. How many biological replicates were used in this analysis; supplementary methods mentions independent replicates but not how many?

17) Fig 4A. How does the red rectangle relate to micro-domains; only one microdomain is shown. Description of arrowheads/triangles is missing/confusing. What are the arrowheads and numbers in the lower panel?

18) Fig 4D Difficult to see the relationship between the points and the interaction names.

19) Fig 5 A left=upper and right = lower?

20) "P-loop" needs clarification in legend.

**Reviewer 3**

**Are you able to assess all statistics in the manuscript, including the appropriateness of statistical tests used?**

Yes, and I have assessed the statistics in my report.

**Comments to author:**

Transparency statement: this review was written by Guillaume Filion (CRG, Barcelona). I was contacted by the Editor on October 9, 2019, I requested 20 days instead of the usual 10, which the Editor agreed on. I submitted my review on November 6, 2019. I worked on it approximately 9 hours. I feel qualified to review the technical aspects of the manuscripts, including the statistics. I declare no conflict of interest.

In the article titled "Insulator-based Loops mediate trans-spreading of H3K27me3 at Micro-domains flanking Euchromatin Genes", Alexandre Heurteau et al. study the interactions between genome folding and histone marks in Drosophila. They perform a Beaf-32 knock down in S2 cells and study the distribution of H3K27me3. They observe that sparse H3K27me3 regions called 'micro-domains' tend to lose signal upon knock down and hypothesize that Beaf-32 indirectly connects those sites to heterochromatin. They later show that the strength of TAD borders is not affected by the knock down and conclude that Beaf-32 must acts by forming specific long-range contacts.

In my opinion, the manuscript needs substantial improvement. There is barely any issue with grammar, but the manuscript is hard to read because of general lack of attention to detail and because the logic and even the meaning of the arguments is difficult to follow.

I have some concerns regarding the scientific quality of the material presented in the manuscript.

Reproducibility issues.

1. The study is presently impossible to reproduce. To make their results reproducible, the authors must release the code that they used for the analysis, together with clear instructions on how to reproduce their results. Ideally, they would provide a virtual machine with the software needed to reproduce their work, together with some test code for their custom code (if any). I will gladly review the code and make sure that it is reproducible in the next round of review. I also request access to the data that was uploaded to GEO and other data sets used by the authors for this study so that I can reproduce the statistics in the figures and in the text. The Materials and Methods section should be amplified with more detail and the reference to methods of other published work should be replaced by references to the corresponding sections in the present manuscript. When this information is available, the manuscript will need another round of review.

Missing important controls.

2. Figure 1E should be plotted again by replacing windows of fixed width around insulator binding sites. The model of the authors shown in Figure 1A and exposed in the title of the section is that micro-domains flank GAF binding sites, not that they are in the same genes (genes can be long so a GAF-bound gene may gain H3K27me3 far away from the GAF binding site).

3. Micro-domains have some properties of statistical noise: their signal is narrow and they tend to disappear in other experimental conditions. Random spikes detected as signal in ChIP-seq are fairly frequent. The authors should rule out that they do not simply pick up statistical spikes. One way to do this is to define micro-domains using one replicate and testing the reduction of H3K27me3 (as per Figure 2B) in another replicate. If the micro-domains are real reproducible signal, there should not be any reduction. In parallel, the authors would run a swap where they define the micro-domains on the Beaf-32 knock down and compare the H3K27me3 with the WT. If the model of the authors is correct, they should obtain fewer micro-domains than in the WT control. Also, the reduction of H3K27me3 (again as per Figure 2B) should be less severe than in the original figure 2B.

4. The authors should show the colocalization of micro-domains with Beaf-32, cohesin, GAF, CTCF and DREF. This association is critical for the argument of the authors, but they do not show it directly. In addition to enrichment values, it is important to also use a representation where the raw numbers appear. There are 1,311 micro-domains, how many are bound by each protein? This information is critical to judge whether the model proposed by the authors is plausible.

5. The authors should show the long-range interactions between micro-domains and insulator sites. The authors have Hi-C data, ChIP-seq data and micro-domains so they can replace the indirect arguments of the second section and of Figure 3 by a direct test on the full genome.

Missing important information.

6. In the text and in Figures 1B and 1C, how are H3K27me3 borders defined?

7. In Figures 1B and 1C, which points are taken for the statistical test? All the points on the right of the coordinate 0? Only a fraction of them?

8. In Figure 1E, what is defined as the binding of an insulator protein in a gene?

9. In Figure 2A, what is the space between the red lines? On my version of the figure, the space before the first line seems smaller. If this is correct, why is that the case? How were the positions of the lines chosen?

10. The authors should show all the data points in Figure 2E. Equivalent Figure 1D shows bars at coordinate 0 but the first pair of bars seems to be missing in Figure 2E.

11. The legend of Figure 5C mentions A/B compartments and refers to the Material and Methods section, however there is no information about how A/B compartments were computed in the Material and Methods nor in the supplementary Material and Methods.

Analyses that need clarification.

12. Figure 4C shows four possible cases and thus represents the data of a 4x2 table. The legend says that the p-values are computed by Fisher's exact test, which works only on 2x2 tables. How were enrichment compared and how was the test performed exactly?

13. Figure 4E shows a plot where there seems to be approximately 2000 TADs. The supplementary Materials and Methods section mentions that the authors found 696 TADs. Was the number of TADs kept constant in the manuscript? Where does the difference come from?

14. There is not enough information in the text and the supplementary material to understand the legend of Figure 5A. The authors should clarify the meaning of LRIs-1, LRIs-2 and LRIs-3.

I also have some concerns regarding the readability of the manuscript. Here are some examples of issues that I encountered during my review.

1. Figure aesthetics. For instance, the legend goes outside the frame in Figure 1D, there seems to be some numbers at the top of Figure 1A, but they are deformed and pixelized to it is impossible to read them. Figure 3D is crossed by packed grid lines (of which two are missing) which make it hard to read the legend and to see the signal.

2. Axis labels. For instance, in Figures 1E and 4C "odds ratio" is spelled "Oddsratio" or "oddsratio". Both are incorrect and actually refer log odds ratio. Capitalization in figure legends seems to be random: "insulator", "profiles" and "chimera" are capitalized as non-first word but not "density" and "level". The axis of Figure 2E reads "Distribution of Insulator sites with decreasing H3K27me3 (Beaf-KD/WT control)" but the legend says "Distribution of sites that harbor a H3K27me3 micro-domain with respect to their distances to heterochromatin borders".

3. Undescribed features. For instance, there is no mention of what the error bars in Figures 1D and 2E represent. The spacing between red lines in Figure 2A is only described as "nucleosomal". The y-axis of the genome browser shots has no scale.

4. Variable spelling. For instance, one can read at different places "from drosophila to humans", "from Drosophila to humans", or "from Drosophila to Human" (both in italics).

5. Buried information. For instance, there is no mention of the model organism in the title, neither in the abstract, neither in the introduction, neither in the results. Beaf-32 is certainly a Drosophila-specific gene but one has to go to the Materials and Methods section to get confirmation that experiments were all performed on Drosophila, and more specifically on S2 cells.

6. Circular statements. For instance, the statement "insulator proteins could contribute to define specific LRIs clustering together distant insulator sites bound by other insulator families like dCTCF or GAF (Liang et al., 2014; Ramirez et al., 2018)" is followed by "Accordingly, an H3K27me3 micro-domain was encountered at the distant GAF insulator sites...". The second statement is in accord with the first only if one assumes that micro-domains are involved in LRIs, which is precisely what the authors set out to demonstrate in this section.

This list is incomplete. Those are mostly style issues but they make the manuscript difficult to read and to review. I encourage the authors to address them for the benefit of their readership.

**Authors Response**

**Point-by-point responses to the reviewers’ comments:**

*We thank all three reviewers for their detailed comments and for positive and constructive criticisms. We have pleased that we could address all of their comments.*

Reviewer #1:

We thank the reviewer for providing with positive comments and constructive criticisms. We now provide our point-by-point response:

1. In Figures 1D and 2E, it is not clear what is actually shown by the horizontal brackets, and why brackets for the BEAF-32 positive and negative borders are overlapping.

*We have removed the horizontal brackets as they would bring some confusion in these two figure panels. The distribution of bins with increasing H3K27me3 levels is shown for borders that harbor a BEAF-32 site, compared to control borders without such binding (as indicated in blue and red, respectively).*

2. I would like to see the reproducibility of the H3K27me3 micro-domains between the ChIP-seq replicates.

*This point was previously addressed by ChIP-qPCR (Figure S2C). We now show our additional analyses of ChIP-seq data that address this point further, as illustrated by the new panels of Figure S6: Figure S6D shows that 60 % of the detected micro-domains can be reproducibly detected between replicates. A more detailed quantification of the signal is shown in Figure S6F left box plot that test if H3K27me3 levels of micro-domains detected in one WT (‘WT1’) condition, are also detected in the replicate experiments (‘WT2’) , or for the mutants (‘mut1’ and ‘mut2’). As shown, the higher levels of H3K27me3 are reproduced in WT2, and mutants largely impair such H3K27me3 levels (as validated by a Wilcoxon pair-wise test). In addition, the right box plot of Figure S6F shows that H3K27me3 levels in micro-domains are significantly higher as compared to control bins of the same sizes, confirming that the specificity of the micro-domains signal.*

3. It is very hard to follow Figure 3D. Please, show positions of the anchor and test fragments at the genomic diagram (similar to the panel A).

*We have now added the primers used for 3C analysis below the corresponding figure panels, as suggested. Please note that the anchor site was designed to probe LRIs with such a distant Beaf-32 site (as suggested by the reviewer).*

4. Significant changes of the contact frequencies measured in 3C should be validated in the reciprocal experiments. Authors should place an anchor primer at the restriction fragment demonstrating a decrease in the interaction frequency with the mio promoter upon the CP190 depletion, and measure its interaction frequency with several test fragments, including the mio promoter.

*The reciprocal experiment assessing interactions from the mio promoter is now shown in cells depleted of CP190 compared to control cells (Supplementary Figure S3D), confirming our previous results. Similar results were obtained when probing interactions in absence of Beaf32 as previously shown by Liang et al. Cuvier, (Figure 2B; Molecular Cell, 53, 672-681).*

5. To state that BEAF-32 KD reduces H3K27me3 level at the studied micro-domain via the disruption of LRI, authors should perform 3C experiments with the anchor primers at the BEAF-32 binding site and micro-domain in the BEAF-32-deficient cells.

*As mentioned above, this is what was shown in the panel of Figure 3A, as now clarified by adding the positions of 3C primers. To address this point in further details, we have tested whether it is a general property of micro-domains to preferentially interact with distant Beaf-32 sites, as now measured genome-wide by Hi-C. The results show preferential LRIs with micro-domains indeed, as now shown in Figure 6D . Of note, the specificity of LRIs between Beaf-32 sites and micro-domains is further validated that probing LRIs in Hi-C from Beaf-32 depleted cells, which alters the preferential LRIs between Beaf-32 sites and micro-domains.*

Reviewer #2:

*We thank this reviewer for providing with very positive comments and helpful criticisms. We now provide our point-by-point response:*

1. The abstract is difficult to read and needs simplifying to concentrate on the main message of the paper; e.g. a) The first sentence introduces TADs and sub-TADS, then the second sentence talks about the association of "small sub-TADs" with insulators: what does this say about large sub-TADs and TADs? The paper seems to be about Beaf-32 flanking TADs rather than sub-TADs or even small sub-TADs. b) it is suggested that mechanisms preventing the rather undefined concept of compartmentalization "leakage" need to be explored but then it turns out that this is not what the paper is about at all.

*We have removed the terms sub-TADs or ‘leakage’, as suggested, to simplify the abstract and keep in focused in the main findings. We thank the reviewer for this suggestion.*

2) Micro-domains are identified on the basis of reduced H3K27me3 in Beaf-32 knockdown so the reduction shown in Fig 2C is not surprising, but why the negative Fold Change in Fig2 B is only seen for micro-domains smaller than 2Kb is unclear.

*Unlike stated, the original identification of putative micro-domains was not performed by comparing wild-type to the depleted condition. Rather, two steps were utilized as now clarified in the Methods section: first a global screen was used, where the micro-domains were obtained by systematic comparison of H3K27me3 relatively to inputs. From then, the levels of H3K27me3 (Log FC) were estimated for such domains, in WT compared to Beaf-32 knockdown (Figure 2B-C). As such, H3K27me3 levels may not systematically decrease upon Beaf-32 knockdown (as opposed to what would have been observed if we had search for differences between WT and Beaf-32 knockdown from the first place). Also, the fold changes of H3K27me3 levels allowed to assess whether the detected micro-domains actually showed significant decrease of H3K27me3 or not, which mostly true for relatively smaller micro-domains (< 2 kb) as previously shown, unlike larger one’s. Our new results confirm that the decreasing levels of H3K27me3 are actually not systematic. Also, a direct comparison of the Log FC in H3K27me3 levels confirm that smaller micro-domains show significantly higher variations compared to larger domains(Supplementary Figure S2D). Also, the 2 kb-window correspond to the bona fide threshold of sizes above which the decrease is not significant as shown (see Supplementary Figure S2E).*

3) Fig 3 indicates two micro-domains in a specific genomic region but, unhelpfully, they are indicated vaguely by arrows rather than definitively by extent. This is made more confusing in Fig 4 where only one micro-domain is indicated at this position. This is important as it affects the interpretation of the 3C result in Fig3D which shows an effect on interaction to the right side of the box but little effect at the interaction point on the left side of the box, which seems to be the closest point to the micro-domain.

*To clarify this issue, the detected micro-domains are now indicated in both panels along with the positions of the primers used for 3C analysis as shown (see Figure 3A, C and D). All primers amplify from the 3’ ends of fragments. Therefore the two arrows correspond a unique fragment that harbor one micro-domain. This 3C fragment is where most significant decreases in long-range interactions (upon Beaf32 or CP190 depletion) are detected (see also Liang et al Cuvier, Mol Cell).*

*To further address whether long-range interactions with distant Beaf-32 sites are a general characteristic of micro-domains, or a simple observation attributed to one locus, we have now probed all long-range interactions as detected by Hi-C, between all Beaf-32 sites and all micro-domains, genome-wide (Figure 6D). This analysis confirm the preferentially interactions of distant Beaf-32 sites with micro-domains. Moreover, Beaf-32 depletion followed by Hi-C impairs such preferential interactions with micro-domains, strongly supporting our previous data and conclusions.*

4) the evidence presented in favour of the model is largely aggregate genomic data that need support from specific instances where clear effects can be observed; e.g. in the chosen selected region in Fig 3 there seems to be no evidence for a specific local interaction between the Beaf-32 site and the micro-domain. It would also be important to show specific local changes in gene expression associated with micro-domains in a number of selected genome regions.

*The 3C data presented actually reflected the interaction between the distant Beaf-32 site (anchor) and the detected micro-domain, as now clarified by showing the primers used for 3C including the anchor site in Figure 3A.*

*Furthermore, the example of long-range interactions shown in Figure 3A actually involves a micro-domain that is nearby the promoter of the mio and crc genes as now shown. Of note, we find that these two genes actually become up-regulated when Beaf32 depletion impairs interactions with the distant Beaf32 site, as now indicated in the text. Also, to test such a linkage of micro-domains with up-regulated genes further, we now provide with a plot showing the genome-wide distribution of all up-regulated or control genes with respect to nearest micro-domains (Figure S6G). This genome-wide analysis confirms that up-regulated genes show a preferential association with a nearby micro-domain compared to control genes, as reflected by mio and crc (Figure 3).*

More detailed issues

1) The title on the manuscript is problematic as there is no evidence that the micro-domains flank euchromatic genes. Generally the use of "flank " in the manuscript is potentially confusing as it is not clear whether it means "occurs on one side of" or "occurs on both sides of". Curiously a different title is given in the cover letter; this title seems better although it's not clear what the domains are distant from.

*The preferential distribution of micro-domains close to (up-regulated) genes has been addressed (Figure S6G; please see our reply to above point 4 and to the other reviewers). Of note, these genes are present in euchromatin.*

2) nomenclature of "trans-spreading". The spreading from large H3K27me3 domains to micro domains appears to be distant spreading in cis rather than "trans". "Distant spreading" would distinguish it from the local spreading at borders.

*We made this change accordingly.*

3) p3 line 19 "Such chromosomal…" this sentence is confusing as it combines two separate phenomena.

*We corrected this accordingly.*

4) p5 line 34 "Such defects are not systematic…" what defects?

*We meant ‘spreading’ defects and change the sentence accordingly.*

5) p5 line 54 "While depletion.." this sentence is confusing as it suggests that depletion of insulator proteins leads to spreading of H3K27me3 into micro-domains; the opposite of what is observed.

*We agree (it was confusing) and made the change accordingly.*

6) p6 line 19 What is the evidence that it is finely-tuned?

*We removed this term.*

7) p7 Heading. "are associated with" might be better than "flank", which is rather confusing.

*We made this change accordingly.*

8) p8 line 22 "sites harbored relatively higher H3K27me3 levels" is confusing as line 44 says the micro-domains had "low H3K27me3 levels".

*We removed this sentence as it brought confusion without adding novel information.*

9)p9 line 12 "Micro-domains are distinct from…heterochromatin domains" presumably some H3K27me3 peaks that are reduced in Beaf-32 knock-down overlap with large H3K27me3 domains. What, if any, is this overlap?

*Micro-domains are defined as domains excluded from large heterochromatin domains independently identified by hidden markov model (HMM), as described.*

10) p9 "epigenetically stable"; is the stability of microdomains addressed?

*Although stability across generation was obviously not addressed, we did show that micro-domains represent sites (or bins) of euchromatin where H3K27me3 levels are relatively more stable compared to other sites/bins taken from euchromatin (with the same distribution of sizes), as shown (Figure S7C). Of note, this analysis assess the stability of such domains as estimated by a K-S test and a Pearson correlation value throughout developmental stages, as described in the Supplementary section.*

11) p10 line 17 not clear what "genes" means here; are only sites overlapping transcription units being analysed? Needs clarification/justification.

*The rationale for such analysis was that we had previously shown that 91.1% of Beaf-32 binding sites were localized near (<1kb) a TSS (Liang et al. Mol Cell). Given that H3K27me3 spreading was observed specifically at Beaf-32 sites, we thus sought to compare such sites with control sites and chose TSSs to ensure that they would be chosen in the same genomic contexts, as now clarified in the Supplementary Methods section. Moreover, we have also verified this result independently of TSSs as now shown in Figure S1F.*

12) p10 line 53 "mio" gene. This is confusing as the coordinates given do not fit with the mio gene located at 2L:2,217,290..2,220,625 according to Flybase (rel6 coordinates). Also the coordinates given in Fig 3 span 44.5kb whereas the Figure indicates 40kb. Additionally the coordinates given in Fig 3 do not make sense with the coordinates given in Figure 4; the right edge of Fig 3 is given as 21,352,228 but Fig4, which apparently extends further to the right, is given as 21,351,215.

*We made these changes accordingly: the coordinates were oriented in reverse; the coordinates 2,307,715 is shown on the right. The TSSs of crc and mio are at 21,308,233 and 21,308, 866 (Crc and crc are two distinct genes).*

13) p11 line 2 why focus the analysis arbitrarily on the 722 micro-domains <1kb from TSS? Needs justification.

*We focused on such domains for the same reason as mentioned above (see point 11), to carefully evaluate the influence of micro-domains on co-localization with other features yet in homogeneous genomic contexts that help comparisons, and also to be able to integrate expression data.*

14) Fig 1. What do the dotted boxes represent? The ones over micro-domains are confusing as they have different dimensions to the indicated microdomains. What micro domains are indicated; the 1,311 list or the 722 list? Do the red bars just indicate micro-domain position or do they represent domain lengths? Blue triangle "on top of genes"; meaning unclear. Is there a significance to the length of brackets in 1E? Why does 1E focus on genes (i.e. transcription units) and how are marks allocated to genes?

*The dotted boxes reflect our original observations that in the same domain, sites flanking heterochromatin show increasing levels in H3K27me3 upon Beaf-KD, while other sites show decreasing levels in the same condition. Also, NormR detect such variations as micro-domains (among 1,311). A detailed characterization of micro-domain length is now provided in Supplementary Figure S2D-E.*

15) Fig 2B. It's not clear to me how micro-domains can have lengths that are smaller than the resolution of ChIP peaks.

*The ’length’ of micro-domains is not comparable to the resolution of ChIP-seq. As described in Supplementary Methods section, we have used several sizes of bins (ranging from 10 to 40 bp) to ensure that micro-domain detection was not an artifact due to a particular choice of bin size. Also, no particular length could be detected when running micro-domain detection after swapping sample with input control.*

16) Fig 3D The sample colours in the Figure do not correspond to the description in the Figure legend. How many biological replicates were used in this analysis; supplementary methods mentions independent replicates but not how many?

*We made the required changes and clarify the number of replicates in the methods.*

17) Fig 4A. How does the red rectangle relate to micro-domains; only one microdomain is shown. Description of arrowheads/triangles is missing/confusing. What are the arrowheads and numbers in the lower panel?

*The red rectangle is the description of the micro-domain by NormR. The arrowheads pinpoint two regions where H3K27me3 levels decrease in the mutant.*

18) Fig 4D Difficult to see the relationship between the points and the interaction names.

*We added arrowheads to show which point correspond to names.*

19) Fig 5 A left=upper and right = lower?

*We corrected this mistake.*

20) "P-loop" needs clarification in legend.

*We made this clarification accordingly in the legend.*

Reviewer #3:

*We thank this reviewer (G. Filion) for providing with thorough and detailed criticisms for how to clarify a number of issues regarding the description of the analyses, including a requirement to provide all necessary materials to reproduce figures. We now provideall R scripts and GEO accession numbers to reproduce all figures. We also provide carefully addressing all criticisms.*

Reproducibility issues.

1. The study is presently impossible to reproduce. To make their results reproducible, the authors must release the code that they used for the analysis, together with clear instructions on how to reproduce their results. Ideally, they would provide a virtual machine with the software needed to reproduce their work, together with some test code for their custom code (if any). I will gladly review the code and make sure that it is reproducible in the next round of review. I also request access to the data that was uploaded to GEO and other data sets used by the authors for this study so that I can reproduce the statistics in the figures and in the text. The Materials and Methods section should be amplified with more detail and the reference to methods of other published work should be replaced by references to the corresponding sections in the present manuscript. When this information is available, the manuscript will need another round of review.

*-We now provide with the codes as R scripts that generated all analyses and figures with the details of our analyses (see also Supplementary Information section). For convenience, R scripts are provided together with data to be able to recompute every figure (each figure is found in a separate folder, and each sub-folder contains the scripts and data for every figure panel), as made accessible through our computer cloud and a CNRS webserver:*

*https://filesender.renater.fr/?s=download&token=b1ca3ffe-0e66-37e5-33b2-783f6f71d582&lang=en*

*(no password required)*

*or*

*https://cloud.biotoul.fr/index.php/s/3eJZ3JroEZxSyxZ*

*password: HeurteauReview20200123*

*-Regarding data, we made the GEO files accessible as GSE130211, that includes replicates of H3K27me3 for mutants and control conditions (GSM3733915, GSM3733916, GSM3733917,GSM3733918, GSM3733919). Additional analyses was performed with our published data (in the same cell line), including expression data or additional data in control or Beaf-KD cells (GSE57168 and GSE52887).*

Missing important controls.

2. Figure 1E should be plotted again by replacing windows of fixed width around insulator binding sites. The model of the authors shown in Figure 1A and exposed in the title of the section is that micro-domains flank GAF binding sites, not that they are in the same genes (genes can be long so a GAF-bound gene may gain H3K27me3 far away from the GAF binding site).

*Figure 1E panel does not quantify variations in H3K27me3 specific of micro-domains. Rather, it was designed to further test if the increase in H3K27me3 levels, as shown in Figure 1C, may be specifically associated with Beaf-32 binding sites.*

*The rationale for such analysis was that we had previously shown that 91.1% of Beaf-32 binding sites were localized near (<1kb) a TSS (Liang et al. Mol Cell). Given that H3K27me3 spreading was observed specifically at Beaf-32 sites, we thus sought to compare such sites with control sites and chose TSSs to ensure that they would be chosen in the same genomic contexts, as now clarified in the Supplementary Methods section. Furthermore, we now added a similar test to further address the reviewer’s suggestion by taking all insulator binding sites (even if not close to a TSS), and then intersecting them with bins ranked by increasing or decreasing levels in H3K27me3, as now shown in Figure S1F.*

*Furthermore, the estimate of H3K27me3 variations was not done on the entire gene but in +/- 1kb windows surrounding TSSs. As such, the variations in H3K27me3 were estimated in the same gene window as for the detection of insulator binding sites or not, which were also scored in the +/- 1 kb window surrounding TSSs, allowing direct enrichment tests.*

3. Micro-domains have some properties of statistical noise: their signal is narrow and they tend to disappear in other experimental conditions. Random spikes detected as signal in ChIP-seq are fairly frequent. The authors should rule out that they do not simply pick up statistical spikes. One way to do this is to define micro-domains using one replicate and testing the reduction of H3K27me3 (as per Figure 2B) in another replicate. If the micro-domains are real reproducible signal, there should not be any reduction. In parallel, the authors would run a swap where they define the micro-domains on the Beaf-32 knock down and compare the H3K27me3 with the WT. If the model of the authors is correct, they should obtain fewer micro-domains than in the WT control. Also, the reduction of H3K27me3 (again as per Figure 2B) should be less severe than in the original figure 2B.

*Figure S6F (left box plot) now shows a box plot comparing H3K27me3 levels of micro-domains detected in one WT (‘WT1’) condition, for the replicate experiments (‘WT2’) , and for the mutants (‘mut1’ and ‘mut2’). As shown, the higher levels of H3K27me3 are reproduced in WT2, and mutants largely impair such H3K27me3 levels (as validated by a Wilcoxon pair-wise test).*

*Figure S6F (right box plot) further shows H3K27me3 levels in micro-domains compared to control bins as suggested (random bins obtained by swapping samples in the same genomic contexts), supporting our results. Additional reproducibility tests were also performed using Fisher exact tests to test whether micro-domains that are impaired upon depletion of Beaf32 were also impaired by the mutant condition (Figure S6A) or to test if such phenomena was reproducibly found in replicates of mutants compared to control (Figure S6D).*

4. The authors should show the colocalization of micro-domains with Beaf-32, cohesin, GAF, CTCF and DREF. This association is critical for the argument of the authors, but they do not show it directly. In addition to enrichment values, it is important to also use a representation where the raw numbers appear. There are 1,311 micro-domains, how many are bound by each protein? This information is critical to judge whether the model proposed by the authors is plausible.

*All numbers are now provided in the Supplementary Tables S1, S2, S3. Further distribution of micro-domains is also shown with respect to TSSs including for up-regulated genes, as now provided in Figure S6G.*

5. The authors should show the long-range interactions between micro-domains and insulator sites. The authors have Hi-C data, ChIP-seq data and micro-domains so they can replace the indirect arguments of the second section and of Figure 3 by a direct test on the full genome.

*We thank the reviewer for this nice suggestion. We now probe long-range interactions of distant Beaf-32 sites with micro-domains, as detected by Hi-C, genome-wide (Figure 6D). This analysis confirm the preferentially interactions of distant Beaf-32 sites with micro-domains. Of interest, Beaf-32 depletion followed by Hi-C impairs such preferential interactions with micro-domains (p-value <1e-4 by Wilcoxon pairwise test), strongly supporting our previous data and conclusions.*

Missing important information.

6. In the text and in Figures 1B and 1C, how are H3K27me3 borders defined?

*This has been clarified in the legend and in the Supplementary Methods section (the processing scripts are also provided).*

7. In Figures 1B and 1C, which points are taken for the statistical test? All the points on the right of the coordinate 0? Only a fraction of them?

*As now mentioned in Figure legend, the statistical test was performed by taking the reads from 0 to +4 kb euchromatin segments flanking heterochromatin (using Wilcoxon pairwise test between Beaf-KD and control).*

8. In Figure 1E, what is defined as the binding of an insulator protein in a gene?

*As now mentioned in Figure legend, it is +/- 1kb from TSS (not the entire gene) where we could show that 91 % of Beaf32 sites were previously encountered (Liang et al., Mol Cell).*

9. In Figure 2A, what is the space between the red lines? On my version of the figure, the space before the first line seems smaller. If this is correct, why is that the case? How were the positions of the lines chosen?

*The spacer are fixed (every 200 bp) as now clarified in the Figure legend. We note however, that there may be no linker between the position ‘0’ and the first nucleosome signal, unlike what is encountered between the next nucleosomes.*

10. The authors should show all the data points in Figure 2E. Equivalent Figure 1D shows bars at coordinate 0 but the first pair of bars seems to be missing in Figure 2E.

*The shift of Figure 1D was a mistake. For Figure 2E, the signal is filtered precisely to avoid mixed effects due to the proximity of borders (as shown in Figure 1D) as for detection of micro-domains.*

11. The legend of Figure 5C mentions A/B compartments and refers to the Material and Methods section, however there is no information about how A/B compartments were computed in the Material and Methods nor in the supplementary Material and Methods.

*We now provide a description of how A/B compartments were computed in the corresponding section of the supplementary Material and Methods.*

Analyses that need clarification.

12. Figure 4C shows four possible cases and thus represents the data of a 4x2 table. The legend says that the p-values are computed by Fisher's exact test, which works only on 2x2 tables. How were enrichment compared and how was the test performed exactly?

*We now provide with a more detailed description of how the test was done in the supplementary method section. Briefly, the Fisher exact test was performed to test an association between the presence of a micro-domain in the flanking euchromatin region (versus absence), depending on the presence or not of a particular arrangement of binding sites.*

13. Figure 4E shows a plot where there seems to be approximately 2000 TADs. The supplementary Materials and Methods section mentions that the authors found 696 TADs. Was the number of TADs kept constant in the manuscript? Where does the difference come from?

*The number of TADs was the same throughout the manuscript except for the GSEA test, as already mentioned in the Figure legend. We now clarify this by providing the details in the Figure legend and in the Supplementary Methods section - data can be reproduced with associated GitHub scripts. For GSEA, we used TADs detected by Wang and colleagues, to improve statistical analysis that helps for such a ranking test.*

14. There is not enough information in the text and the supplementary material to understand the legend of Figure 5A. The authors should clarify the meaning of LRIs-1, LRIs-2 and LRIs-3.

*The figure legend was completed as suggested.*

Concerns regarding the readability of the manuscript.

1. Figure aesthetics. For instance, the legend goes outside the frame in Figure 1D, there seems to be some numbers at the top of Figure 1A, but they are deformed and pixelized to it is impossible to read them. Figure 3D is crossed by packed grid lines (of which two are missing) which make it hard to read the legend and to see the signal.

*This has been fixed. We apologize for the too-limited resolution of the figures provided in our original submission.*

2. Axis labels. For instance, in Figures 1E and 4C "odds ratio" is spelled "Oddsratio" or "oddsratio". Both are incorrect and actually refer log odds ratio. Capitalization in figure legends seems to be random: "insulator", "profiles" and "chimera" are capitalized as non-first word but not "density" and "level". The axis of Figure 2E reads "Distribution of Insulator sites with decreasing H3K27me3 (Beaf-KD/WT control)" but the legend says "Distribution of sites that harbor a H3K27me3 micro-domain with respect to their distances to heterochromatin borders".

*All items have been fixed. Actually, results for insulators sites are shown in 2E, while 2F show the distribution of micro-domains.*

3. Undescribed features. For instance, there is no mention of what the error bars in Figures 1D and 2E represent. The spacing between red lines in Figure 2A is only described as "nucleosomal". The y-axis of the genome browser shots has no scale.

*This has been fixed.*

4. Variable spelling. For instance, one can read at different places "from drosophila to humans", "from Drosophila to humans", or "from Drosophila to Human" (both in italics).

*This has been fixed.*

5. Buried information. For instance, there is no mention of the model organism in the title, neither in the abstract, neither in the introduction, neither in the results. Beaf-32 is certainly a Drosophila-specific gene but one has to go to the Materials and Methods section to get confirmation that experiments were all performed on Drosophila, and more specifically on S2 cells.

*This has been fixed.*

6. Circular statements. For instance, the statement "insulator proteins could contribute to define specific LRIs clustering together distant insulator sites bound by other insulator families like dCTCF or GAF (Liang et al., 2014; Ramirez et al., 2018)" is followed by "Accordingly, an H3K27me3 micro-domain was encountered at the distant GAF insulator sites...". The second statement is in accord with the first only if one assumes that micro-domains are involved in LRIs, which is precisely what the authors set out to demonstrate in this section.

*This has been fixed.*

**Second round of review**

**Reviewer 2**

The authors have amended the manuscript to address many of my concerns. The data now presented in Figure S6G provides support for the general relevance of the micro-domains for gene regulation. I agree with reviewer 3 that the micro-domains “have some properties of statistical noise” and I note that reviewer 2 also raises the issue of reproducibility and I agree this is an important issue for the authors to address. In the revised version the authors present an analysis of the replicates (FigS6F) that to my mind does provide support for the reproducibility of the micro-domains.

However, there remain several problems with the Figures e.g.

a) Figure 1. The top line says “chromosome 2R: 5,762,600 .. 5,747,600” suggesting that the genome sequence is represented backwards whereas the barely legible map coordinates appear to go from 5762kb to 5774kb upwards from left to right.

In addition the description of the figure is still problematic. The legend mentions “The triangle” but there are two triangles in the Figure. Despite the rebuttal comment “The dotted boxes reflect our original observations….” there is still no mention in the figure legend of what the dashed rectangles represent, presumably the two on the right refer to the micro-domains (red rectangles at bottom) but why are they are a different size from the red domains. The legend says “The ChIP-seq of GAF (blue triangle) is indicated on top of genes.”; it’s not clear what this means and doesn’t seem to reflect the position.

b) Figure 3. Again the coordinates in the top line are descending, chr2L: 21,352,228-21,307,715 which is odd. The genes are also difficult to identify as the authors do not seem to be using current gene annotation. It is not clear what annotation they are using; in the methods the ChIP reads were mapped to Drosophila genome dm3 (which is Release 5 from 2006). The current release, Release6, came out in 2014 and the authors should really be using this or give a justification why not. The authors should use current Flybase gene names. What do the asterisks mean after the gene names? The crc gene is current, the gene the authors call “mio” conflicts with the current Flybase mio which, as I mentioned in my first review, is elsewhere, and the gene called “tsp39D” is presumably Tsp39D. The gene called “mio” in the figure is presumably the Flybase gene Mondo, which has a synonym Mio. However the gene order is Mondo…crc…Tsp39D which conflicts with the Figure. Overall, it is difficult to work out what is going on here.

c) In Fig 4 the coordinates at the top are ascending but the gene order is the same as Fig 3.

**Reviewer 3**

Transparency statement: this review was written by Guillaume Filion (CRG, Barcelona). I received the invitation to review the second version of the article on January 29, 2020. I notified the Editor on February 12, 2020 that I would need extra time to finish the review. On February 28, 2020 I asked for extra time again and I turned in my review on March 8, 2020. I worked 12-13 hours on this review. I declare no conflict of interest.

In the previous round, the other reviewers and I raised some concerns about the lack of clarity of the manuscript, calling for an in-depth reorganization and for substantial rewriting. This version presents only shallow modifications and the pervasive lack of clarity remains just the same. Before explaining this more in detail, I would like to highlight some major improvements.

1. The data and the code are available. The authors have opened the GEO record before publication, which speaks highly of their commitment to make the study reproducible. The code was provided to me, and I hope that they will make it available when the article is published. Saying that it was possible to reproduce the manuscript with “just one click” is an overstatement because I could not even run the code (it depends on a long list of R packages), but the code was in the repository and I could read it.

2. The authors have convinced me that micro-domains are not a statistical artifact. The result of the test they set up in Supplementary Figure 6F is clear.

Overall, the manuscript remains tedious to read, to the extent that even after 20+ hours of work I still do not understand how the data were handled and I still cannot emit a proper judgement about the validity of the analyses. This would take much more work, which neither the readers nor I can be expected to provide.

The issues mostly boil down to lack of attention to details, and it is difficult for me to present them in a way that can both show the magnitude of the situation and help the authors fix it. I have divided the content into sections where I first discuss general issues with data and statistical practice, then I list unaddressed issues from the previous round, and finally give a list of points that need clarification. I have to add that addressing all the comments would only allow the manuscript to be properly reviewed. For me, the manuscript is not ready for publication and it needs a complete recast, including tighter analyses and a reshaped flow.

SECTION 1: ISSUES WITH STATISTICS AND DATA HANDLING

1. The data are not consistent throughout the manuscript. To give an example, the authors initially find 1,311 micro-domains but quickly select the subset of 722 micro-domains within 1 kb of a TSS. The reason for this is not clear. A little later (page 14 line 19), the authors mention 901 “micro-domains identified in wild-type and that were lost in Beaf32 depleted cells”. This is yet a third definition of micro-domains, also not explained.

2. The data sets are inconsistent throughout the manuscript. The first round of review revealed that the authors change TAD data set in the middle of the manuscript “to improve statistical analysis that helps for such a ranking test” (quoted from the rebuttal). A one-off change of data set to improve the statistics may be appropriate in some exceptional circumstances, but this would need to be highlighted and justified at length. This is otherwise considered bad practice by most statisticians.

3. The GEO record GSE130211 is incorrectly annotated.

The summary field reads “We used the technnology of ChIP-sequencing to analyze changes in H3K27 levels in Drosophila schneider S2 depleted of endogenous Beaf32 and expressing either mutated insulator looping mutants (mut-Beaf32 ) as compared cells depleted of endogenous protein and expressing wild-type Beaf32”. There is a typo at “technology”, an ill-defined reference to “mut-Beaf32” with a bracket-style issue and a missing “to” after “compared”. In addition, the text is misleading because H3K27me3 is referred to as “H3K27”.

The overall design field reads “SupT1 T-ALLL were treated for 2 days with DMSO or GSI and the different factors of the Notch repressive Complex were ChIPed”. This has obviously nothing to do with the current study and must be the result of an unfortunate copy-paste.

SECTION 2: UNADDRESSED ISSUES FROM THE PREVIOUS ROUND

Previous issue 1.

4. The authors should show the colocalization of micro-domains with Beaf-32, cohesin, GAF, CTCF and DREF. This association is critical for the argument of the authors, but they do not show it directly. In addition to enrichment values, it is important to also use a representation where the raw numbers appear. There are 1,311 micro-domains, how many are bound by each protein? This information is critical to judge whether the model proposed by the authors is plausible.

In this new version there is still no figure showing that micro-domains overlap with GAF and CTCF as claimed in the title of the first section. The main text points to Figure 1E (page 8 line 22 and page 9 line 36), which in the words of the authors in the rebuttal “does not quantify variations in H3K27me3 specific of micro-domains”.

Previous issue 2.

12. Figure 4C shows four possible cases and thus represents the data of a 4x2 table.

The legend says that the p-values are computed by Fisher's exact test, which works

only on 2x2 tables. How were enrichment compared and how was the test performed

exactly?

The more detailed description of the authors reads “For statistical tests probing LRIs variations between Beaf32-KD and control cells (Wang et al., 2018), we used fisher exact test after genome-wide ranking of TADs depending on LRI 1/2/3s.” This explanation does not clarify which 2x2 table was used for the Fisher test.

Looking at the code provided by the authors, I could get the answer: the class “None” is used as reference and is recycled over the 4 cases, so that the odds ratio and the p-values are for the enrichment relative to this category (where Beaf32 is neither on the left nor on the right border). The rest of the manuscript seems to use the same approach, but the class used as a reference is never specified (e.g., Figure 1E).

Previous issue 3.

1. Figure aesthetics. For instance, the legend goes outside the frame in Figure 1D, there seems to be some numbers at the top of Figure 1A, but they are deformed and pixelized to it is impossible to read them. Figure 3D is crossed by packed grid lines (of which two are missing) which make it hard to read the legend and to see the signal.

It does not look like the issue is addressed, and this does not look like a resolution issue either. At maximum zoom, all the features look smooth, except some overly compressed numbers over the gene track that (I assume) read 5762 kb, 5764 kb… The horizontal grid lines in panel 3D also do not look like a resolution issue either. If they are not present on the version of the authors, then the image has been seriously modified during the upload.

Previous issue 4.

2. Axis labels. For instance, in Figures 1E and 4C "odds ratio" is spelled "Oddsratio" or "oddsratio". Both are incorrect and actually refer log odds ratio. Capitalization in figure legends seems to be random: "insulator", "profiles" and "chimera" are capitalized as non-first word but not "density" and "level". The axis of Figure 2E reads "Distribution of Insulator sites with decreasing H3K27me3 (Beaf-KD/WT control)" but the legend says "Distribution of sites that harbor a H3K27me3 micro-domain with respect to their distances to heterochromatin borders".

The x axis of Figure 2D reads “Normalized density”, that of Figure 2F reads “Normalized Density”. The random capitalization patterns in axis labels continue throughout the figures.

Previous issue 5.

3. Undescribed features. For instance, there is no mention of what the error bars in Figures 1D and 2E represent. The spacing between red lines in Figure 2A is only described as "nucleosomal". The y-axis of the genome browser shots has no scale.

There are at least three types of error bars used in scientific documents, and there are multiple ways to plot each of them. See for instance see

https://www.nature.com/articles/nmeth.2659?error=cookies_not_supported&code=44fdae6b-c248-415c-95ab-bc0b79307a5e. The figure legend has to specify whether the bars represent the standard deviation, the standard error of the mean, a confidence interval or something else. If they are standard deviation, say, it has to be clear whether the standard deviation is one full bar (between tick marks) or one half bar (from the middle to a tick mark).

SECTION 3: ISSUES WITH LACK OF CLARITY

These issues are presented in the order of the text. Those are only the things I could not understand for sure, however long I tried. The main reason is style, which is exaggeratedly vague and complex. To show what I mean, take this random sentence from the beginning of the text:

Systematic measurements of H3K27me3 levels depending on both Beaf32 binding and on Beaf32 depletion (‘Beaf-KD’) highlighted a relatively modest yet significant impact on H3K27me3 levels as compared to siRNA treated control cells (Figure 1B-C; p-value of 1e-4)(Supplementary Figure S1A).

Most readers would better understand something along the lines of “The levels of H3K27me3 decrease more at sites where Beaf32 is lost upon knock-down (Figure 1B-C, Supplementary Figure S1A, Student’s unpaired two-tailed t test, n=412, P < 0.0001).” The authors are of course free to choose the style that suits them, but it is important to remember that style is supposed to make scientific texts easier to understand.

1. Page 3 Line19: TADs notably favor specific long-range contacts between distant sites and regulatory elements that localize within the same topological unit.

It is unclear what “favor” means in this context. I suppose it is a mis-translation of “favoriser”, which should have been translated to “promote” or equivalent.

2. Page 3 Line 46: TAD insulators may restrict LRIs with sites pertaining to adjacent TADs.

Do the authors mean “belonging to” instead of “pertaining to”?

3. Page 7 Line 12: Insulator proteins including CTCF often bind to sites flanking heterochromatin domains from Drosophila to human (Cuddapah et al., 2009; Emberly et al., 2008), as illustrated for Beaf32 (Figure 1A).

The motivation for bringing Beaf32 in the picture is awkward. From the title, the paragraph seems to be about dCTCF and GAF, but it's almost entirely about Beaf32 for reasons that are hard to follow. Perhaps a better option is to cut this paragraph in two, and rename the first part something like “Loss of Beaf32 leads to loss of H3K27me3 micro-domains”.

4. Page 7 Line 29: Moreover, the increase in H3K27me3 levels was specific over total histone H3 reads (Figure 1B-C), and it may account for global gene down-regulation (Supplementary Figure S1B)(Lhoumaud et al., 2014).

It is unclear what “specific over total histone H3” means. The last statement is ambiguous: it could mean that there is a global gene regulation, explained by the increase of H3K27me3 or that H3K27me3 explains the global pattern of down-regulation. The supplementary figure suggests the second, but it is still ambiguous: the results are shown in a table with log-odds ratio combined with a Fisher test, but there there is no 2x2 contingency table anywhere. On what numbers the Fisher test was performed is not clear from the context.

5. Page 7 Line 51: Our results showed that the influence of Beaf32 was not drastic, raising the possibility that additional factors are required.

It is unclear what those additional factors are required for. From the context, it is probably for blocking the spread of H3K27me3, in which case other factors are not necessary but sufficient, so the meaning is the opposite.

6. Page 9 line 37: Analyzing the distribution of such insulator sites with decreasing H3K27me3 levels confirmed their relative enrichment from 5 to 30 kb distances from a Beaf32 site as shown (Figure 2E).

Using “confirmed” suggests that this idea was already put forward. In reality this observation has no logical connection with the preceding statement that micro-domains “reflect” the loss of H3K27me at GAF/dCTCF sites. To support the first claim, the authors should show that micro-domains co-localize with GAF/dCTCF sites but they seem unwilling to do so (see unaddressed points).

7. Page 9 line 41: Similarly, H3K27me3 micro-domains preferentially localized in euchromatin bordered a Beaf32 site (Figure 2F).

Figure 2F does not show this.

8. Page 10 line 2: We hypothesized that weak TADs unable to restrain H3K27me3 within such topological unit might lead to micro-domain formation.

It is unclear why the authors state this hypothesis, immediately propose an alternative and focus exclusively on the alternative. This is even more confusing that the first hypothesis reappears in the following section. It is not clear what the authors try to prove or disprove, and how this is connected with the main point of the manuscript.

9. Page 10 line 5: Alternatively, insulator proteins could contribute to define specific LRIs with distant dCTCF or GAF (Liang et al., 2014; Ramirez et al., 2018).

It is unclear what the alternative hypothesis really is because it states an accepted fact in the field. Perhaps the authors mean that micro-domains appear when GAF/dCTCF form a long-range contact with a heterochromatic site bound by Beaf32?

10. Page 10 line 24: Accordingly, genome-wide analysis showed an enrichment of sites with most significant decreases in H3K27me3 levels providing co-localization with CP190 (Figure 3B, upper matrix; p-value of 1-4).

The meaning of “providing” is unclear, and I cannot make sense of this sentence. Figure 3B shows a reduced overlap between the sites bound by CP190-bound and those that lose H3K27me3. Also, the sentence starts with “accordingly” but the preceding statement just says that Beaf32 depends on CP190. The argumentation is hard to follow here.

11. Page 10 line 29: Accordingly, the enrichment of CP190 sites harboring an increase in H3K27me3 was lost when co-localized with GAF (Figure 3B, upper matrix; p-value of 0.1)

or dCTCF, in stark contrast to what was detected when it co-localizes with Beaf32 (Supplementary Figure S3A-B).

The chain of “accordingly” adds to the confusion. The preceding sentence mentioned a decrease of H3K27me3 at CP190-bound sites, whereas this one mentions an increase. Also, the p-value is not specified on Figure 3B but it shows one star: the convention for a p-value between 0.05 and 0.01. It is hard to follow what the authors wish to show here.

12. Page 11 line 34: This analysis shows that Beaf32 binds to the borders of the most robust TADs genome-wide (Figure 4D)...

It is unclear what Figure 4D shows. For instance, what are the horizontal and vertical lines that cross the scatter plot? Also, there are 5 blue dots, 9 red dots and no color legend. The meaning of this figure escapes me entirely.

13. Page 11 line 44: ...as tested using gene set enrichment analysis (GSEA) (Figure 4E; see Material and Methods).

There are two panels in Figure 4E, but the legend does not explain their difference. Why there are 2000 TADs in one panel and 1000 in the other is not commented.

14. Page 12 line 1: Insulator binding sites not only bracket TADs, they also define one of the most significant sites in terms of LRIs with distant sites in the genome...

The meaning of “one of the most significant” is unclear. There are obviously more than one sites and there is no explanation of what a significant LRI is.

15. Page 12 line 7: Such properties are notably detected in presence of insulator proteins...

It is unclear which properties are referred to.

16. Page 12 line 12: Of note, these are unique features specifically detected with insulator protein sites, and not found for control sites as shown by global assessment of LRIs as a function of protein binding (Figure 4D, see y-axis).

It is not obvious how to compare the scatter plot of Figure 4D (itself unclear) to the aggregated Hi-C map of Figure 5A.

17. Page 12 line 12: Inspection of the characteristic eigen’s value reflecting euchromatin/heterochromatin into distinct A/B compartments (see Materials and

Methods) showed that micro-domains may not be totally separated from B compartments

(Figure 5C).

A/B compartment scores are not the eigenvalues of the loci but their coordinates on the first eigenvector. The y-axis of Figure 5C is thus incorrect. The argument seems to rests on the idea that the first quartile of this score is below 0 for micro-domains. To my knowledge, 0 is not a special value that separates A compartments from B compartments.

18. Page 13 line 29: Additional aggregation of Hi-C data highlighted loops/LRIs between Beaf32 and distant GAF/dCTCF/CP190 insulator sites in control cells, which is impaired upon Beaf32 depletion (Figure 6A).

It is unclear what is impaired, especially because the only word in singular of the sentence is “aggregation”. From the context and from Figure 6A, it seems that loops are impaired.

19. (Page 13 line 65): There is no mention of Figure 6D in the text.

20. Page 14 line 36: Averaged CP190 profiles were decreased by the mutants (Figure 7B)...

Figure 7B shows H3K27me3 signal so it is hard to see how this fits the description in the text.

21. Page 14 line 36: Of interest, micro-domains contributed to control the expression of nearby genes that become up-regulated upon depletion of Beaf32 (Figure 7D; p-value of 1e-4; see also Supplementary Figure S6G).

The box plots of Figure 7D do not look plausible to me. The category “All TSSs” seems to stop around the value 1 on the upper side. In the absence of whiskers and outliers, this means that not a single gene has a log-fold change greater than 1. Yet, the genes with a micro-domain represented by the blue box plot seem to have log-fold change values mostly above 1. They are certainly part of “All TSSs”, so how was the data treated exactly, and how is this possible at all? Also note that the y-axis label hides the minus sign of -10 and thus makes the figure wrong.

**Authors Response**

**Point-by-point responses to the reviewers’ comments:**

Reviewer #2:

The authors have amended the manuscript to address many of my concerns. The data now presented in Figure S6G provides support for the general relevance of the micro-domains for gene regulation. I agree with reviewer 3 that the micro-domains “have some properties of statistical noise” and I note that reviewer 2 also raises the issue of reproducibility and I agree this is an important issue for the authors to address. In the revised version the authors present an analysis of the replicates (FigS6F) that to my mind does provide support for the reproducibility of the micro-domains.

However, there remain several problems with the Figures e.g.

a) Figure 1. The top line says “chromosome 2R: 5,762,600 .. 5,747,600” suggesting that the genome sequence is represented backwards whereas the barely legible map coordinates appear to go from 5762kb to 5774kb upwards from left to right. In addition the description of the figure is still problematic. The legend mentions “The triangle” but there are two triangles in the Figure.

Despite the rebuttal comment “The dotted boxes reflect our original observations….” there is still no mention in the figure legend of what the dashed rectangles represent, presumably the two on the right refer to the micro-domains (red rectangles at bottom) but why are they are a different size from the red domains. The legend says “The ChIP-seq of GAF (blue triangle) is indicated on top of genes.”; it’s not clear what this means and doesn’t seem to reflect the position.

*We made all of these corrections.*

b) Figure 3. Again the coordinates in the top line are descending, chr2L: 21,352,228-21,307,715 which is odd.

The genes are also difficult to identify as the authors do not seem to be using current gene annotation. It is not clear what annotation they are using; in the methods the ChIP reads were mapped to Drosophila genome dm3 (which is Release 5 from 2006). The current release, Release6, came out in 2014 and the authors should really be using this or give a justification why not. The authors should use current Flybase gene names.

*We made these corrections. We now provide all data using current gene names from Dm6. We now provide the updated list of Dm6 genes in Table S2.*

What do the asterisks mean after the gene names?

*The asterisks mark the genes being down-regulated (*) or up-regulated (**), as now explained in the corresponding Figure legend.*

The crc gene is current, the gene the authors call “mio” conflicts with the current Flybase mio which, as I mentioned in my first review, is elsewhere, and the gene called “tsp39D” is presumably Tsp39D. The gene called “mio” in the figure is presumably the Flybase gene Mondo, which has a synonym Mio. However the gene order is Mondo…crc…Tsp39D which conflicts with the Figure. Overall, it is difficult to work out what is going on here.

*We corrected these annotations (using Dm6 annotations).*

c) In Fig 4 the coordinates at the top are ascending but the gene order is the same as Fig 3.

*We made this correction.*

Reviewer #3: Transparency statement: this review was written by Guillaume Filion (CRG, Barcelona). I received the invitation to review the second version of the article on January 29, 2020. I notified the Editor on February 12, 2020 that I would need extra time to finish the review. On February 28, 2020 I asked for extra time again and I turned in my review on March 8, 2020. I worked 12-13 hours on this review. I declare no conflict of interest.

In the previous round, the other reviewers and I raised some concerns about the lack of clarity of the manuscript, calling for an in-depth reorganization and for substantial rewriting. This version presents only shallow modifications and the pervasive lack of clarity remains just the same. Before explaining this more in detail, I would like to highlight some major improvements.

1. The data and the code are available. The authors have opened the GEO record before publication, which speaks highly of their commitment to make the study reproducible. The code was provided to me, and I hope that they will make it available when the article is published.

Saying that it was possible to reproduce the manuscript with “just one click” is an overstatement because I could not even run the code (it depends on a long list of R packages), but the code was in the repository and I could read it.

*We now provide with a GitHub repository including all scripts pertaining to this manuscript (the same scripts as previously provided to reviewers). Such scripts were made public at:*

*https://github.com/CuvierLab/K27me3_mdom_spreading/tree/master/src*

2. The authors have convinced me that micro-domains are not a statistical artifact. The result of the test they set up in Supplementary Figure 6F is clear.

*We thank the reviewer for noticing these positive points.*

SECTION 1: ISSUES WITH STATISTICS AND DATA HANDLING

1. The data are not consistent throughout the manuscript. To give an example, the authors initially find 1,311 micro-domains but quickly select the subset of 722 micro-domains within 1 kb of a TSS. The reason for this is not clear.

*We sought to test whether the 1,311 micro-domains identified have any detectable influence on gene expression. For this reason, we tested if the 1,311 micro-domains could be associated with nearby genes as encountered for 722 micro-domains (within 1 kb). The second rationale is because the Hi-C data was at a resolution of 1 kb (bins) allowing to keep the same criteria.*

A little later (page 14 line 19), the authors mention 901 “micro-domains identified in wild-type and that were lost in Beaf32 depleted cells”. This is yet a third definition of micro-domains, also not explained.

*There is only one definition of micro-domains. As already mentioned in the first round of revision, micro-domains were identified by systematic scoring of H3K27me3 levels compared to input. As such, the finding that 901/1,301 micro-domains are actually impaired in Beaf32 depleted cells actually supported the idea that they corresponded to a signal specific signal.*

2. The data sets are inconsistent throughout the manuscript. The first round of review revealed that the authors change TAD data set in the middle of the manuscript “to improve statistical analysis that helps for such a ranking test” (quoted from the rebuttal). A one-off change of data set to improve the statistics may be appropriate in some exceptional circumstances, but this would need to be highlighted and justified at length. This is otherwise considered bad practice by most statisticians.

*The GSEA analysis was actually performed following the same criteria: long-range interactions were measured within the limits of the same larger TADs (the same 696 contiguous TADs of a median size ~110Kbp), as in other panels. Yet as represented in Figure 4B and 5B, what was tested actually required to use the smaller 2000 TADs identified that do correspond to such repressive heterochromatin TADs, unlike larger one’s (as shown in several independent studies and notably Szabo et al). We have clarified this in the Supplementary Methods:*

*« To optimize statistical ranking test by Gene set enrichment analysis (GSEA), we utilized high-resolution Hi-C detecting approximately 2,000 TADs throughout the genome (Wang et al., 2018), which corresponded well to smaller, repressive TADs as indicated in our scheme (Figure 4B). Of note however,long-range interactions were still estimated for bins localized within the same larger TADs (among the same 696 contiguous TADs of a median size ~110Kbp), as in all other analyses. »*

3. The GEO record GSE130211 is incorrectly annotated.

*The mistakes in the GEO were edited (validated by GEO). We thank the reviewer for noticing us.*

SECTION 2: UNADDRESSED ISSUES FROM THE PREVIOUS ROUND

Previous issue 1.

4. The authors should show the colocalization of micro-domains with Beaf-32, cohesin, GAF, CTCF and DREF. This association is critical for the argument of the authors, but they do not show it directly. In addition to enrichment values, it is important to also use a representation where the raw numbers appear. There are 1,311 micro-domains, how many are bound by each protein? This information is critical to judge whether the model proposed by the authors is plausible.

In this new version there is still no figure showing that micro-domains overlap with GAF and CTCF as claimed in the title of the first section. The main text points to Figure 1E (page 8 line 22 and page 9 line 36), which in the words of the authors in the rebuttal “does not quantify variations in H3K27me3 specific of micro-domains”.

*The complete list of micro-domains associated with genes and insulator binding sites were provided in the Tables S1-S2, as now better described by providing a detailed description for each column, in the legend of these tables. Of note, we had already shown the profile of CP190 at micro-domains (yet there has been a mistake in the legend of the y-axis of Figure 7B, which did show CP190 profiles). We further extend ou analysis by providing box plots showing the levels of the three major insulator proteins involved, in Supplementary Figure S6E, confirming the higher binding levels of these insulator factors, as validated by Wilcoxon tests compared to control sites (random sites in euchromatin).*

Previous issue 2.

12. Figure 4C shows four possible cases and thus represents the data of a 4x2 table.

The legend says that the p-values are computed by Fisher's exact test, which works

only on 2x2 tables. How were enrichment compared and how was the test performed

exactly?

*The enrichment test was performed by testing if a combination is individually enriched compared to the absence of the sites (first lane), as now stated in the legend and indicated by an accolade in the figure. The results thus show that each combination is actually enriched compared to no binding.*

The more detailed description of the authors reads “For statistical tests probing LRIs variations between Beaf32-KD and control cells (Wang et al., 2018), we used fisher exact test after genome-wide ranking of TADs depending on LRI 1/2/3s.” This explanation does not clarify which 2x2 table was used for the Fisher test.

*We agree: this statement has nothing to do with the 2x2 table of Figure 4C.*

Looking at the code provided by the authors, I could get the answer: the class “None” is used as reference and is recycled over the 4 cases, so that the odds ratio and the p-values are for the enrichment relative to this category (where Beaf32 is neither on the left nor on the right border). The rest of the manuscript seems to use the same approach, but the class used as a reference is never specified (e.g., Figure 1E).

*Yes, this is correct. We clarify this in all panels.*

Previous issue 3.

1. Figure aesthetics. For instance, the legend goes outside the frame in Figure 1D, there seems to be some numbers at the top of Figure 1A, but they are deformed and pixelized to it is impossible to read them. Figure 3D is crossed by packed grid lines (of which two are missing) which make it hard to read the legend and to see the signal.

*The corresponding panels were corrected. We now provide with a high resolution version of the figures after verifying the aesthetic issues mentioned. The grid lines in Figure 3D is actually because of a break in the scale of the y-axis.*

Previous issue 4.

2. Axis labels. For instance, in Figures 1E and 4C "odds ratio" is spelled "Oddsratio" or "oddsratio". Both are incorrect and actually refer log odds ratio. Capitalization in figure legends seems to be random: "insulator", "profiles" and "chimera" are capitalized as non-first word but not "density" and "level". The axis of Figure 2E reads "Distribution of Insulator sites with decreasing H3K27me3 (Beaf-KD/WT control)" but the legend says "Distribution of sites that harbor a H3K27me3 micro-domain with respect to their distances to heterochromatin borders ». The x axis of Figure 2D reads “Normalized density”, that of Figure 2F reads “Normalized Density”. The random capitalization patterns in axis labels continue throughout the figures.

*We made these corrections.*

Previous issue 5.

3. Undescribed features. For instance, there is no mention of what the error bars in Figures 1D and 2E represent. The spacing between red lines in Figure 2A is only described as "nucleosomal". The y-axis of the genome browser shots has no scale.

The figure legend has to specify whether the bars represent the standard deviation, the standard error of the mean, a confidence interval or something else. If they are standard deviation, say, it has to be clear whether the standard deviation is one full bar (between tick marks) or one half bar (from the middle to a tick mark).

*We made each of these corrections.*

SECTION 3: ISSUES WITH LACK OF CLARITY

Systematic measurements of H3K27me3 levels depending on both Beaf32 binding and on Beaf32 depletion (‘Beaf-KD’) highlighted a relatively modest yet significant impact on H3K27me3 levels as compared to siRNA treated control cells (Figure 1B-C; p-value of 1e-4)(Supplementary Figure S1A).

Most readers would better understand something along the lines of “The levels of H3K27me3 decrease more at sites where Beaf32 is lost upon knock-down (Figure 1B-C, Supplementary Figure S1A, Student’s unpaired two-tailed t test, n=412, P < 0.0001).” The authors are of course free to choose the style that suits them, but it is important to remember that style is supposed to make scientific texts easier to understand.

*We modified this sentence accordingly.*

1. Page 3 Line19: TADs notably favor specific long-range contacts between distant sites and regulatory elements that localize within the same topological unit.

*We changed this accordingly.*

2. Page 3 Line 46: TAD insulators may restrict LRIs with sites pertaining to adjacent TADs.

Do the authors mean “belonging to” instead of “pertaining to”?

*We changed this accordingly.*

3. Page 7 Line 12: Insulator proteins including CTCF often bind to sites flanking heterochromatin domains from Drosophila to human (Cuddapah et al., 2009; Emberly et al., 2008), as illustrated for Beaf32 (Figure 1A).

*We corrected the first sentence accordingly.*

4. Page 7 Line 29: Moreover, the increase in H3K27me3 levels was specific over total histone H3 reads (Figure 1B-C), and it may account for global gene down-regulation (Supplementary Figure S1B)(Lhoumaud et al., 2014).

*We clarified this sentence.*

It is unclear what “specific over total histone H3” means. The last statement is ambiguous: it could mean that there is a global gene regulation, explained by the increase of H3K27me3 or that H3K27me3 explains the global pattern of down-regulation. The supplementary figure suggests the second, but it is still ambiguous: the results are shown in a table with log-odds ratio combined with a Fisher test, but there there is no 2x2 contingency table anywhere. On what numbers the Fisher test was performed is not clear from the context.

*We clarified this in the text and the legend of Supplementary Figure 1B.*

5. Page 7 Line 51: Our results showed that the influence of Beaf32 was not drastic, raising the possibility that additional factors are required.

It is unclear what those additional factors are required for. From the context, it is probably for blocking the spread of H3K27me3, in which case other factors are not necessary but sufficient, so the meaning is the opposite.

*We clarified this sentence accordingly.*

6. Page 9 line 37: Analyzing the distribution of such insulator sites with decreasing H3K27me3 levels confirmed their relative enrichment from 5 to 30 kb distances from a Beaf32 site as shown (Figure 2E).

Using “confirmed” suggests that this idea was already put forward. In reality this observation has no logical connection with the preceding statement that micro-domains “reflect” the loss of H3K27me at GAF/dCTCF sites. To support the first claim, the authors should show that micro-domains co-localize with GAF/dCTCF sites but they seem unwilling to do so (see unaddressed points).

*We made this correction (the co-localization has been addressed in above ‘point 4’/previous issue section).*

7. Page 9 line 41: Similarly, H3K27me3 micro-domains preferentially localized in euchromatin bordered a Beaf32 site (Figure 2F).

Figure 2F does not show this.

*We clarified this accordingly.*

8. Page 10 line 2: We hypothesized that weak TADs unable to restrain H3K27me3 within such topological unit might lead to micro-domain formation.

It is unclear why the authors state this hypothesis, immediately propose an alternative and focus exclusively on the alternative. This is even more confusing that the first hypothesis reappears in the following section. It is not clear what the authors try to prove or disprove, and how this is connected with the main point of the manuscript.

*TADs represent insulation from neighbor domains. A ‘weaker’ TADs (or a weak insulation) may then lead to promiscuous LRIs between adjacent TADs. Our point was solely to test if there is a link between TAD strength and micro-domains, which is not found; yet we still like to raise the question and show the results, as we believe that it is interesting, even though the answer is negative.*

9. Page 10 line 5: Alternatively, insulator proteins could contribute to define specific LRIs with distant dCTCF or GAF (Liang et al., 2014; Ramirez et al., 2018).

It is unclear what the alternative hypothesis really is because it states an accepted fact in the field. Perhaps the authors mean that micro-domains appear when GAF/dCTCF form a long-range contact with a heterochromatic site bound by Beaf32?

*We made this correction. The term ‘alternative’ was to distinguish this statement from the other possibility (in previous sentence, related to weak TADs), not to say that this alternative was unlikely.*

10. Page 10 line 24: Accordingly, genome-wide analysis showed an enrichment of sites with most significant decreases in H3K27me3 levels providing co-localization with CP190 (Figure 3B, upper matrix; p-value of 1-4).

The meaning of “providing” is unclear, and I cannot make sense of this sentence. Figure 3B shows a reduced overlap between the sites bound by CP190-bound and those that lose H3K27me3. Also, the sentence starts with “accordingly” but the preceding statement just says that Beaf32 depends on CP190. The argumentation is hard to follow here.

*We made this change accordingly.*

11. Page 10 line 29: Accordingly, the enrichment of CP190 sites harboring an increase in H3K27me3 was lost when co-localized with GAF (Figure 3B, upper matrix; p-value of 0.1)

or dCTCF, in stark contrast to what was detected when it co-localizes with Beaf32 (Supplementary Figure S3A-B).

The chain of “accordingly” adds to the confusion. The preceding sentence mentioned a decrease of H3K27me3 at CP190-bound sites, whereas this one mentions an increase. Also, the p-value is not specified on Figure 3B but it shows one star: the convention for a p-value between 0.05 and 0.01. It is hard to follow what the authors wish to show here.

*We have removed this confusing sentence.*

12. Page 11 line 34: This analysis shows that Beaf32 binds to the borders of the most robust TADs genome-wide (Figure 4D)...

It is unclear what Figure 4D shows. For instance, what are the horizontal and vertical lines that cross the scatter plot? Also, there are 5 blue dots, 9 red dots and no color legend. The meaning of this figure escapes me entirely.

*We now provide these information in the legend of the figure. The lines represent the limit for the top-5% of Hi-C interactions among all Hi-C bins.*

13. Page 11 line 44: ...as tested using gene set enrichment analysis (GSEA) (Figure 4E; see Material and Methods).

There are two panels in Figure 4E, but the legend does not explain their difference. Why there are 2000 TADs in one panel and 1000 in the other is not commented.

*It was indicated in the panel: left is all TADs, and right the TADs flanked by Beaf32, as now better explained in the legend of the figure.*

14. Page 12 line 1: Insulator binding sites not only bracket TADs, they also define one of the most significant sites in terms of LRIs with distant sites in the genome...

The meaning of “one of the most significant” is unclear. There are obviously more than one sites and there is no explanation of what a significant LRI is.

*We made this correction to be more explicit; please also see above our reply to point 12 that shows that insulator binding sites are among the sites with highest interaction levels in 3D.*

15. Page 12 line 7: Such properties are notably detected in presence of insulator proteins...

It is unclear which properties are referred to.

*We remove this term.*

16. Page 12 line 12: Of note, these are unique features specifically detected with insulator protein sites, and not found for control sites as shown by global assessment of LRIs as a function of protein binding (Figure 4D, see y-axis).

It is not obvious how to compare the scatter plot of Figure 4D (itself unclear) to the aggregated Hi-C map of Figure 5A.

*The analysis is the same: Figure 4D shows the values obtained in the squares as indicated in Figure 5A, as now better explained in the legend.*

17. Page 12 line 12: Inspection of the characteristic eigen’s value reflecting euchromatin/heterochromatin into distinct A/B compartments (see Materials and Methods) showed that micro-domains may not be totally separated from B compartments (Figure 5C).

A/B compartment scores are not the eigenvalues of the loci but their coordinates on the first eigenvector. The y-axis of Figure 5C is thus incorrect. The argument seems to rests on the idea that the first quartile of this score is below 0 for micro-domains. To my knowledge, 0 is not a special value that separates A compartments from B compartments.

*We now better describe the method accounting for how A/B compartments can be reflected by the sign of the eigenvalues (please see Supplementary Methods), as previously done by Fortin and colleagues (Fortin JP, Hansen KD. Reconstructing A/B compartments as revealed by Hi-C using long-range correlations in epigenetic data. Genome Biol. 2015;16(1):180).*

*The procedure is also detailed in the Supplementary Methods, with a link Github/PROCESSING_SCRIPTS/COMPARTMENT_COMPUTATION into https://github.com/CuvierLab/K27me3_mdom_spreading/tree/master/src). This process allowed us to identify A and B compartments and followed by attribution of signs to the eigen vector (such that every A or B compartments has a positive or negative value, respectively (hence the Y-axis correspond to the value of eigen vector).*

18. Page 13 line 29: Additional aggregation of Hi-C data highlighted loops/LRIs between Beaf32 and distant GAF/dCTCF/CP190 insulator sites in control cells, which is impaired upon Beaf32 depletion (Figure 6A).

It is unclear what is impaired, especially because the only word in singular of the sentence is “aggregation”. From the context and from Figure 6A, it seems that loops are impaired.

*We corrected this.*

19. (Page 13 line 65): There is no mention of Figure 6D in the text.

*Thank you for noticing this.*

20. Page 14 line 36: Averaged CP190 profiles were decreased by the mutants (Figure 7B)...

Figure 7B shows H3K27me3 signal so it is hard to see how this fits the description in the text.

*There was a mistake in the y-axis: the average profile is actually the ChIP-seq of CP190 (hence some misunderstanding with the reviewer that we had not done this analysis).*

21. Page 14 line 36: Of interest, micro-domains contributed to control the expression of nearby genes that become up-regulated upon depletion of Beaf32 (Figure 7D; p-value of 1e-4; see also Supplementary Figure S6G).

The box plots of Figure 7D do not look plausible to me. The category “All TSSs” seems to stop around the value 1 on the upper side. In the absence of whiskers and outliers, this means that not a single gene has a log-fold change greater than 1. Yet, the genes with a micro-domain represented by the blue box plot seem to have log-fold change values mostly above 1. They are certainly part of “All TSSs”, so how was the data treated exactly, and how is this possible at all? Also note that the y-axis label hides the minus sign of -10 and thus makes the figure wrong.

*The issue was that our control TSSs was designed to filter out de-regulated genes. Yet as noticed, the filter showed some issues (apparently not working for most down-regulated genes) and was removed. However the p-values were correctly calculated without filters, between the indicated groups of gene flanking micro-domains and control genes (without micro-domain), or for genes flanking a Beaf32 site. This has now been corrected and explained in the legend and figure.*

**Third round of review**

**Reviewer 1**

I carefully read the new version of the manuscript, the authors' answers to the questions of the reviewer. I think that all my original points have been properly addressed.
